# Multimodal fusion of brain imaging and proteomics reveals a brain–body pathway linking depression and metabolic dysfunction

**DOI:** 10.1017/S003329172610436X

**Published:** 2026-05-13

**Authors:** Zhengxu Lian, Zhaowen Liu, Huaxin Fan, Jiazheng Wang, Kai Zhang, Yu Liu, Nanyu Kuang, Gechang Yu, Wei Cheng, Benjamin Becker, Barbara J. Sahakian, Trevor W. Robbins, Vince D. Calhoun, Jing Sui, Xinran Wu, Jie Zhang, Jianfeng Feng

**Affiliations:** 1 Institute of Science and Technology for Brain-inspired Intelligence, Fudan University, Shanghai, China; 2 School of Computer Science, Northwestern Polytechnical University, Xian, China; 3 School of Computer Science and Technology, East China Normal University, Shanghai, China; 4 National Institutes of Health, Baltimore, USA; 5 The Chinese University of Hong Kong, Hong Kong; 6 Department of Psychology, University of Hong Kong, Hong Kong; 7 Department of Psychiatry, University of Cambridge, UK; 8 Georgia Institute of Technology, USA; 9 State Key Laboratory of Cognitive Neuroscience and Learning, Beijing Normal University, Beijing, PR China; 10 School of Psychology, Shanghai Jiao Tong University, Shanghai, China

**Keywords:** depression, fMRI, multimodal fusion, plasma protein

## Abstract

**Background:**

Depression is associated with pathological dysregulations affecting both the brain and the body, with the latter being reflected in plasma proteins. While plasma protein signatures of depression have been increasingly recognized, a holistic examination of interactions with brain features is lacking.

**Methods:**

Leveraging data from 3,966 UK Biobank participants, we identified a multimodal neuroimaging-plasma protein component of depression (NeuroPro-Dep) by integrating plasma proteins and five brain modalities via an ICD-10 diagnosis-constrained multimodal fusion approach.

**Results:**

Notably, NeuroPro-Dep demonstrates detectable associations with depression symptoms across datasets from diverse populations, underscoring its clinical potential. This capability is anchored in its five brain modalities alterations, including hippocampal atrophy, reduced cortical sensorimotor network functional connectivity, and impaired internetwork structural connectivity of the frontoparietal network. The multimodal neuroimaging-derived plasma protein modality of NeuroPro-Dep is enriched in metabolic pathways, as further supported by association analysis linking this modality to body mass index (BMI), type 2 diabetes, and other metabolic indicators. Crucially, two-step Mendelian randomization analysis revealed that the NeuroPro-Dep plasma protein modality exerts a causal effect on depression through BMI (plasma protein to BMI: or=0.28, p=0.035; BMI to depression: or=1.14, p=4.37×10^−11^).

**Conclusions:**

Overall, this study underscores metabolic dysfunction as a bridge between brain changes, depression, and physical diseases, while providing a novel multimodal biological signature and valuable insights that may inform future treatment strategies.

## Introduction

Depression is one of the most common and severe mental disorders, with high prevalence rates in the population, affecting an estimated 15% (Andrade et al., 2003). Its primary symptoms include low mood, anhedonia, sleep disturbances, cognitive impairment, and suicidal tendencies, accompanied by high rates of recurrence, self-harm, and mortality, making it one of the most significant public health challenges worldwide. Current treatment options for depression are often associated with significant side effects and require a prolonged onset of action (Marwaha et al., 2023). Therefore, there is an urgent need for a better in-depth understanding of the pathological pathways underlying the disorder.

An increasing number of studies have highlighted the significance of brain–body interactions (Sammons et al., 2024), particularly in the etiopathology of psychiatric disorders (Gold et al., 2020). Numerous studies have identified a strong association between depression and various clusters of physical diseases, including diabetes, metabolic syndrome, cardiovascular diseases, cancer, and osteoporosis. According to epidemiological analyses and Mendelian randomization (MR) studies, these physical diseases share a bidirectional relationship with depression, further supporting the need to integrate both into a unified framework (Berk et al., 2023). Integrating physical diseases and depression into a unified framework has recently facilitated the development of theoretical models, advancing the understanding of the underlying mechanisms of depression. The development of these theories is based on clinical data revealing a high level of comorbidity between the two conditions (Berk et al., 2023) and their bidirectional influence, which can further exacerbate comorbid conditions (Berk et al., 2023; Luppino et al., 2010). They may share biological pathways, including the hypothalamic-pituitary-adrenal axis (Stetler & Miller, 2011), inflammation (Osimo et al., 2020), gut microbiome, genetics (Wray et al., 2018), and metabolism (Hagenaars et al., 2020).

According to the central dogma, proteins act as mediators of cellular functions and represent the endpoints of gene expression. They play a crucial role determining the functioning of the aforementioned biological pathways. Although some studies have explored the relationship between plasma proteins and depression (Kang et al., 2025), the majority have been limited by small sample sizes and a narrow range of protein types, constraining the reliability of their findings. More importantly, these studies primarily perform univariate analyses of the associations between plasma proteins and depression phenotypes, without considering the critical role that brain structure and function play in these biological pathways. This oversight persists despite substantial evidence demonstrating the association between alterations in brain structure and function with depression (Schmaal et al., 2017; Shen et al., 2025; Zhu et al., 2024), as well as extensive research highlighting the complex high-order correlations and cross-modal interactions between the brain and other organs mediated through various biological pathways (Tian, Cole, Bullmore, & Zalesky, 2024; B. Zhao et al., 2023). Incorporating diverse brain structural and functional imaging phenotypes into the exploration of biological pathways underlying depression can help bridge the gap between physical mechanisms and mental symptoms (Tian et al., 2024). Thus, we employed the MCCAR+JICA (Qi et al., 2018) multimodal data fusion method to explore previously unidentified plasma protein–brain covariation patterns in depression. This approach enables the identification of key connecting pathways, facilitating a more holistic perspective in understanding the etiology of depression and advancing its treatment.

In summary, we hypothesize the existence of an unexplored multimodal covariation pattern involving depression phenotypes, plasma proteins, as well as brain structure and function alterations. This pattern may inform biological pathways that deepen our understanding of the etiology and mechanisms underlying the comorbidity of depression and physical diseases. To achieve this, we employed multimodal data fusion techniques, which surpass unimodal analysis in capturing the complex relationships among these features and extracting highly informative insights (Calhoun & Sui, 2016). By fusing 2,920 plasma proteins, five brain structural and functional modalities, and ICD-10 depression diagnoses as a constraint, we derived the multimodal neuroimaging-plasma protein covariation component of depression (NeuroPro-Dep) from data involving 3,966 individuals from the UK Biobank. We validated the generalizability of this component in the UK Biobank validation dataset and two external independent datasets. Finally, we identified key biological pathways, genetic associations, and phenotypic links to the plasma protein modality of this component and explored its causal relationship with depression.

## Methods

### Participants

This study utilized data from the UK Biobank cohort. Ethical approval was obtained from the Human Biology Research Ethics Committee at the University of Cambridge (Cambridge, UK). All participants provided informed consent (https://biobank.ctsu.ox.ac.uk/crystal/field.cgi?id=200).

The discovery and UKB validation cohorts for this study were obtained from the UK Biobank. The discovery cohort comprised 4,759 participants who completed both plasma protein sampling and brain imaging. Of these, 306 depression patients and 3,606 healthy controls were identified based on ICD-10 diagnostic criteria. Following previous work (N. Cai et al., 2020), healthy controls were determined by excluding all participants with any ICD-10-coded mental disorders, whereas depression patients were selected by excluding individuals with other potential confounding mental disorders and ensuring that the onset date of depression preceded the data collection date for all modalities.

For the validation cohorts in the UK Biobank, we divided participants into a plasma protein validation cohort and a brain imaging validation cohort. The plasma protein validation cohort included 36,991 participants who completed plasma protein sampling, excluding those already included in the discovery cohort. Similarly, the brain imaging validation cohort was identified using the same criteria, resulting in 28,645 participants.

### Multimodal fusion

Multimodal fusion approach is essential in this study, as it enables the discovery of higher-order correlations and cross-modal interactions between plasma proteins and the brain – a direct mediator of depression, which have not been well characterized previously.

The data used for multimodal fusion were obtained from the UK Biobank and included five neuroimaging features, encompassing morphological measures as well as SC and functional connectivity (FC), plasma proteins, and ICD-10 depression diagnosis labels. Additional details are provided in the Supplementary Materials (eMethods: Modalities for Multimodal Fusion).

Before the data fusion stage, basic covariates, including sex, age, and head motion, were included in the model. Further details are provided in the Supplementary Materials (eMethods: Covariates).

Multimodal fusion was first applied to the neuroimaging and plasma protein data from the discovery cohort using the MCCAR+jICA method (http://trendscenter.org/software/fit) (Qi et al., 2018). Specifically, in this study, the ICD-10 depression diagnosis labels served as the reference signal to jointly decompose the five neuroimaging data tables (X1–X5) and the plasma protein data table (X6). This resulted in the identification of a multimodal neuroimaging–proteomic covariation component of depression (NeuroPro-Dep) characterized by subject-wise loading vectors ( 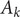 ) for each modality and corresponding modality-specific feature importance vectors ( 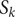 ) within each modality. The supervised fusion method ensured that 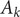 achieved maximal correlation under depression diagnosis’s constraint, as described in Equation 1.

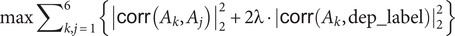

Subject-wise loading vectors 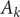 of the identified NeuroPro-Dep must not only demonstrate significant associations between and the ICD-10 depression diagnosis labels but also exhibit linked significant changes across modalities between healthy controls and depression patients, ensuring group discriminative properties.

The robustness of this multimodal component was further validated through a permutation test (see Supplementary Figure S1), which confirmed that the observed associations were highly unlikely to have arisen by chance, indicating that NeuroPro-Dep captures a genuine and biologically relevant multimodal pattern rather than random noise.

### Association analysis using RDS-4 scale

In the association analysis, the RDS-4 scale (derived from field IDs 2050–2080 by directly summing the scores) was used to reflect the severity of depressive symptoms in patients. For the brain imaging validation cohort, RDS-4 scores recorded during the 2.0 follow-up visit were used, whereas baseline records were utilized for the plasma protein validation cohort.

**Figure 1.**
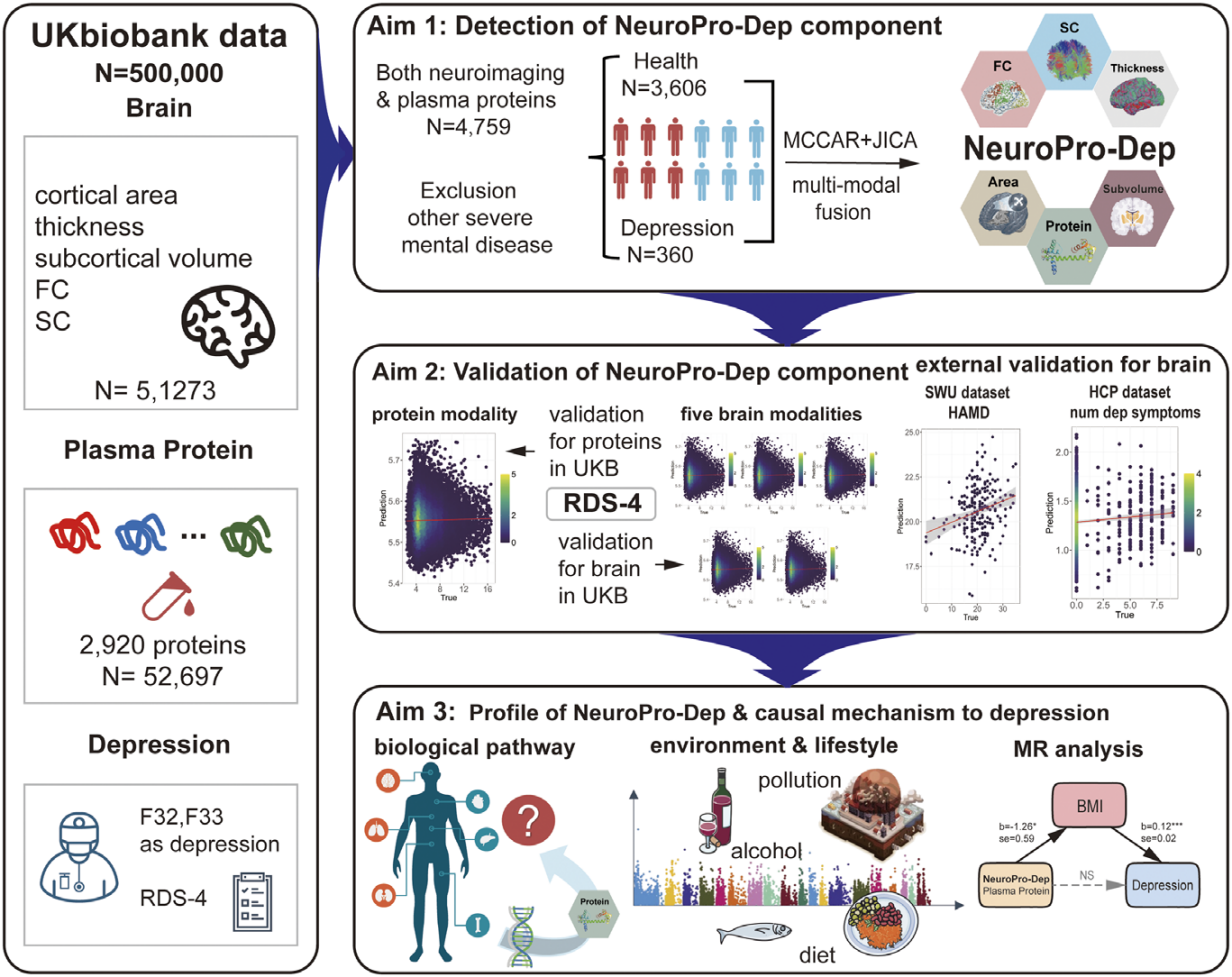
Comparison of Loadings and spatial maps of NeuroPro-Dep across five neuroimaging modalities and plasma proteins between healthy controls and depression patients. *Note*: Panels (a–f) present the spatial maps of the multimodal neuroimaging–plasma protein covariation component of depression (NeuroPro-Dep) for each modality (left part of each subplot) and boxplots comparing the subject loadings of healthy controls and depression patients (right part of each subplot). In the spatial maps, z-values reflect the contribution of each feature within each modality. In panels (d) and (e), the heatmaps summarize network-level structural and functional connectivity by showing the density of suprathreshold connections (i.e. the number of edges exceeding the positive or negative Z-value threshold normalized by the total possible edges) between each pair of cortical networks and subcortical regions. Network identities are indicated by the color bar legend along the axes: visual (VIS, dark purple), sensorimotor (SOM, blue), dorsal attention (DAN, dark green), ventral attention (VAN, magenta), limbic (LIM, light yellow), frontoparietal (FPN, orange), default mode (DMN, red), and subcortical regions (SUB, dark brown). In the spatial maps, red indicates higher values in healthy controls compared to patients, while blue indicates the opposite. Panel (e) uses a Manhattan plot to represent the spatial map of 2,920 plasma proteins. Gray points indicate plasma proteins with |z| < 3, while red and blue points indicate higher or lower contributions in healthy controls, respectively, as described above. In the boxplots, consistent with spatial maps, red represents the distribution of loadings for healthy controls, while blue represents that for depression patients. (* p < .05, ** p < .01, SC, structural connectivity, FC, functional connectivity, subvolume, subcortical volume).

Following previous work (Qi et al., 2022; Sui et al., 2018), we constructed linear regression models that integrated both spatially localized and system-level modality information derived from NeuroPro-Dep. For each modality, two spatial features were obtained from the NeuroPro-Dep–estimated feature-importance vector ( 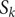 ) by applying an absolute-value threshold and averaging the subject-specific data within regions showing positive and negative importance, respectively (yielding two features per subject; 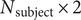 ). These 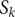 maps, shown in the left panels of Figure 1, illustrate the spatial distribution of feature importance for each modality. In addition to these spatial features, the corresponding subject loading vectors ( 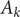 ) – representing each subject’s overall expression strength of the latent modality component – were also included as predictors. Incorporating both 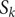 -derived and 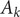 -derived terms enabled the model to jointly capture regional, direction-specific effects and global system-level modulation associated with the outcome variable. The same modeling logic was consistently applied in the *Validation of NeuroPro-Dep in External Datasets* and *Genetic and Phenotype-Wide Association Analysis of NeuroPro-Dep* sections to ensure methodological coherence across analyses. Detailed information on model construction and feature selection is provided in the Supplementary Materials (eMethods: Association Analysis Using RDS-4 Scale).

### Validation of NeuroPro-Dep in external datasets

To evaluate the performance of this multimodal neuroimaging–proteomic covariation component of depression (NeuroPro-Dep) in external datasets, we analyzed the association between the brain imaging modality and depressive symptoms using the HCP dataset and the SWU depression dataset. Detailed information can be found in the Supplementary Materials (eMethods: Validation of NeuroPro-Dep in External Datasets).

### Exploring the biological pathways associated with the plasma protein modality of NeuroPro-Dep

In exploring the biological pathways associated with the plasma protein modality of this multimodal neuroimaging–proteomic covariation component of depression (NeuroPro-Dep), we first evaluated the pathways involving proteins with a threshold of |Z| > 3. These significant proteins were annotated for biological pathways and tissue enrichment using the gene2func module available on the FUMA platform (https://fuma.ctglab.nl/).

To analyze whether these significant proteins differ in biological pathways from proteins directly associated with depression diagnoses, we referred to methods from previous literature to get those direct associated proteins (Kang et al., 2025). The method for identifying this set of directly associated proteins (referred to as cox proteins in the results) is detailed in the Supplementary Materials (eMethods: Identification of Directly Associated Proteins).

To compare the two groups of proteins, we employed the WGCNA package in R to construct a co-expression network based on the expression similarity of the 2,920 plasma proteins (Langfelder & Horvath, 2008). Modules were subsequently identified by clustering proteins with similar expression patterns.

### Genetic and phenotype-wide association analysis of NeuroPro-Dep

To investigate which environmental and lifestyle factors, genetic risks, as well as physical diseases, are associated with the plasma protein modality of this multimodal neuroimaging–proteomic covariation component of depression (NeuroPro-Dep), we conducted a phenome-wide association analysis and polygenic risk score analyses for various physical diseases and biomarkers. Furthermore, Cox regression models were applied to evaluate the association of this component with future risk of a range of physical diseases, aiming to elucidate the comorbidity risk profile of depression. Detailed methodologies are provided in the Supplementary Materials (eMethods: Phenotype-Wide Association Analysis of NeuroPro-Dep, eMethods: Polygenic Risk Score Association Analysis of NeuroPro-Dep, eMethods: Association with Future Physical Disease Risk).

### Mendelian randomization

We employed a two-step MR approach (Carter et al., 2021) to investigate the causal relationships between the multimodal neuroimaging–proteomic covariation component of depression (NeuroPro-Dep) plasma protein modality and body mass index (BMI) as well as depression. The GWAS for the NeuroPro-Dep plasma protein loadings was conducted using quality-controlled genotyping data from Caucasian participants in the UK Biobank. We maintained consistency with the previous section by using the plasma protein validation cohort for population selection, adjusting for covariates including age, sex, the first 20 principal components of genetic data, and relevant plasma protein-related covariates, with the GWAS analysis conducted using PLINK 2 (Chang et al., 2015). The BMI GWAS data were sourced from the Genetic Investigation of Anthropometric Traits consortium (Yengo et al., 2018). Depression data were derived from the latest large-scale multicenter GWAS (N = 1,639,572) conducted by the Psychiatric Genomics Consortium (Adams et al., 2025), excluding UKB data to minimize bias from participant overlap.

For the MR analysis of the NeuroPro-Dep plasma protein modality and BMI, we selected SNPs with a genome-wide significance threshold (p < 1 × 10^−9^) as instrumental variables, applying clumping (1,000 kb distance, maximum linkage disequilibrium r^2^ of .01). The primary analytic method was inverse variance weighted, with sensitivity analyses conducted using weighted median, weighted mode, MR Egger, and simple mode approaches. Additionally, we employed the MR-PRESSO package to identify and exclude SNPs with horizontal pleiotropy. Finally, we conducted tests for horizontal pleiotropy, leave-one-out analysis, and heterogeneity; Details of the results can be found in the Supplementary Materials (eTable). The same methodology was applied to analyze the causal effects of BMI and the NeuroPro-Dep plasma protein modality on depression. To ensure the absence of reverse causality between the plasma proteins, BMI, and depression, we performed the Steiger test, with results provided in the Supplementary Materials. MR analyses were performed using the TwoSampleMR R package (Hemani et al., 2018) and MR-PRESSO R package (Verbanck, Chen, Neale, & Do, 2018).

## Results

### Significant differences in NeuroPro-Dep between healthy and depressed groups

**Figure 2.**
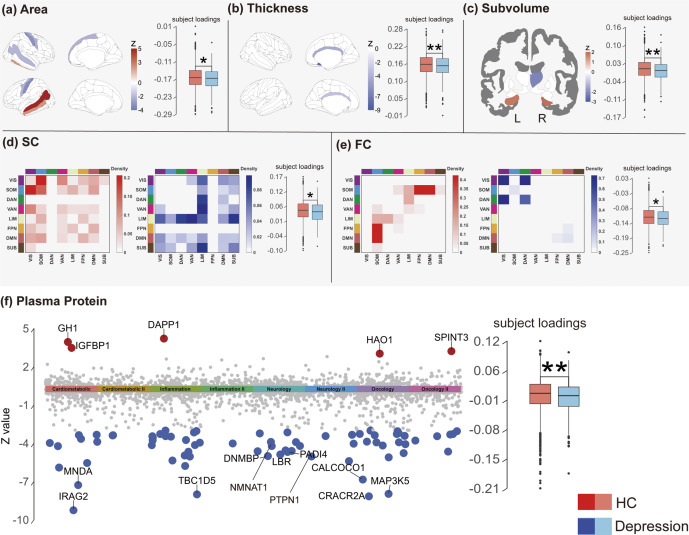
Study overview. *Note*: UKB data: Five neuroimaging modalities and plasma proteins were selected, comprising six modalities in total, with ICD-10 depression diagnoses used as the reference signal for data fusion. Aim 1: A total of 3,966 participants with overlapping plasma protein and neuroimaging data from the UK Biobank were included, excluding individuals with other psychiatric disorders. The curated dataset was fed into the MCCAR+jICA multimodal data fusion model to derive the multimodal neuroimaging–plasma protein covariation component of depression (NeuroPro-Dep). Aim 2: Validation of NeuroPro-Dep was performed on a subset of the UKB dataset that did not include participants used in the multimodal fusion analysis and further validated in two external datasets. Aim 3: Exploration of the biological pathways associated with NeuroPro-Dep plasma protein modality, its relationships with various phenotypes, and causal mechanism to depression.

Our goal is to identify biological networks underlying the covariation of multimodal neuroimaging features and plasma proteins expression, which can serve as biomarkers to elucidate the mechanisms of depression. To this end, we applied the MCCAR+jICA data fusion method (Qi et al., 2018; see Methods for detail). Using ICD-10 depression diagnoses as a reference signal, we conducted fusion analyses on UKB participants who underwent both plasma protein profiling and neuroimaging assessments, as shown in Figure 2, Aim 1. Five representative MRI-based modalities – cortical surface area, cortical thickness, subcortical volume, cortical structural connectivity (SC), and cortical FC – together with plasma proteomic features, were input into the multimodal fusion model. These MRI/fMRI modalities were selected to comprehensively capture complementary brain structural and functional alterations most relevant to depression, covering both macrostructural (area, thickness, subcortical volume) and network-level (SC and FC) organization. This combination also reflects the most frequently reported MRI-derived markers of depression in large-scale neuroimaging studies (Dutt et al., 2022; Gallo et al., 2023). The reliability of these markers is underscored by recent findings across thousands of participants, which robustly identify widespread cortical thickness reduction (Shen et al., 2025) and global FC disruptions linked to fundamental molecular and synaptic gene expression patterns (Zhu et al., 2024). Thus, it provides a balanced and biologically meaningful foundation for cross-modal integration with proteomic data.

One of the joint components derived from the model exhibited significant associations with ICD-10 depression diagnoses across modalities, as confirmed through permutation testing described in Methods, and was termed the multimodal neuroimaging–proteomic covariation component of depression (NeuroPro-Dep). The modality-specific spatial maps of this component are shown in the left parts of subplots in Figure 1. We found that NeuroPro-Dep exhibited high loadings in brain regions such as the sensorimotor cortex, inferior parietal lobule, bilateral temporal lobes, cingulate gyrus, subcallosal gyrus, and subcortical structures like the thalamus and hippocampus, indicating structural and functional alterations in these regions in depression patients compared to healthy controls. Specifically, patients showed increased cortical surface area in the sensorimotor cortex and decreased FC between sensorimotor network and other brain networks, decreased surface area in the left superior temporal sulcus, increased surface area of the right superior temporal sulcus. Structural connectivity showed a clear pattern characterized by weakened connections within the visual–sensorimotor pathway and reduced frontoparietal connectivity, accompanied by strengthened subcortical interactions. Finally, the subcallosal gyrus exhibited increased cortical thickness, while subcortical regions showed decreased hippocampal volume and increased right thalamic volume.

Importantly, the plasma protein modality highlighted several proteins with abnormal expression in depression patients, such as IRAG2, PTPN1, DNMBP, NMNAT1, CRACR2A, and GH1, which are involved in biological processes related to metabolic dysregulation, neuroprotection, neurodegenerative diseases, and immune responses. To validate whether this proteomic component can distinguish between healthy individuals and patients, we performed two-sample t-tests on the loadings of the control and patient groups. The results for each modality, shown in the right panels of the subplots in Figure 1, all revealed significant group differences (surface area: t = 2.00, p = .045*; cortical thickness: t = 2.62, p = .0087**; SC: t = 2.34, p = .019*; FC: t = 2.11, p = .035*; plasma protein: t = 2.77, p = 5 × 10^−3^**; subcortical volume: t = 3.14, p = .017*). Across all modalities, higher subject loadings were consistently observed in healthy controls compared with depression patients, reflecting a coherent direction of association. These findings indicate that NeuroPro-Dep represents a coherent joint multimodal biological signature associated with depression diagnosis, capable of distinguishing between healthy individuals and patients.

### Associative strength and generalizability of NeuroPro-Dep

To further confirm whether this multimodal neuroimaging–proteomic covariation component of depression (NeuroPro-Dep) is associated with current depressive symptoms in a broader UKB population, we constructed multiple linear models using the features of each modality in NeuroPro-Dep. This was to ensure that NeuroPro-Dep is indeed associated with depressive symptoms and demonstrates good generalizability as shown in Figure 2, Aim 2. These models were applied to participants with available data for the corresponding modality, excluding all UKB participants involved in the multimodal fusion analysis. See Section “Methods” for the linear model construction and participant selection for each modality.

**Figure 3.**
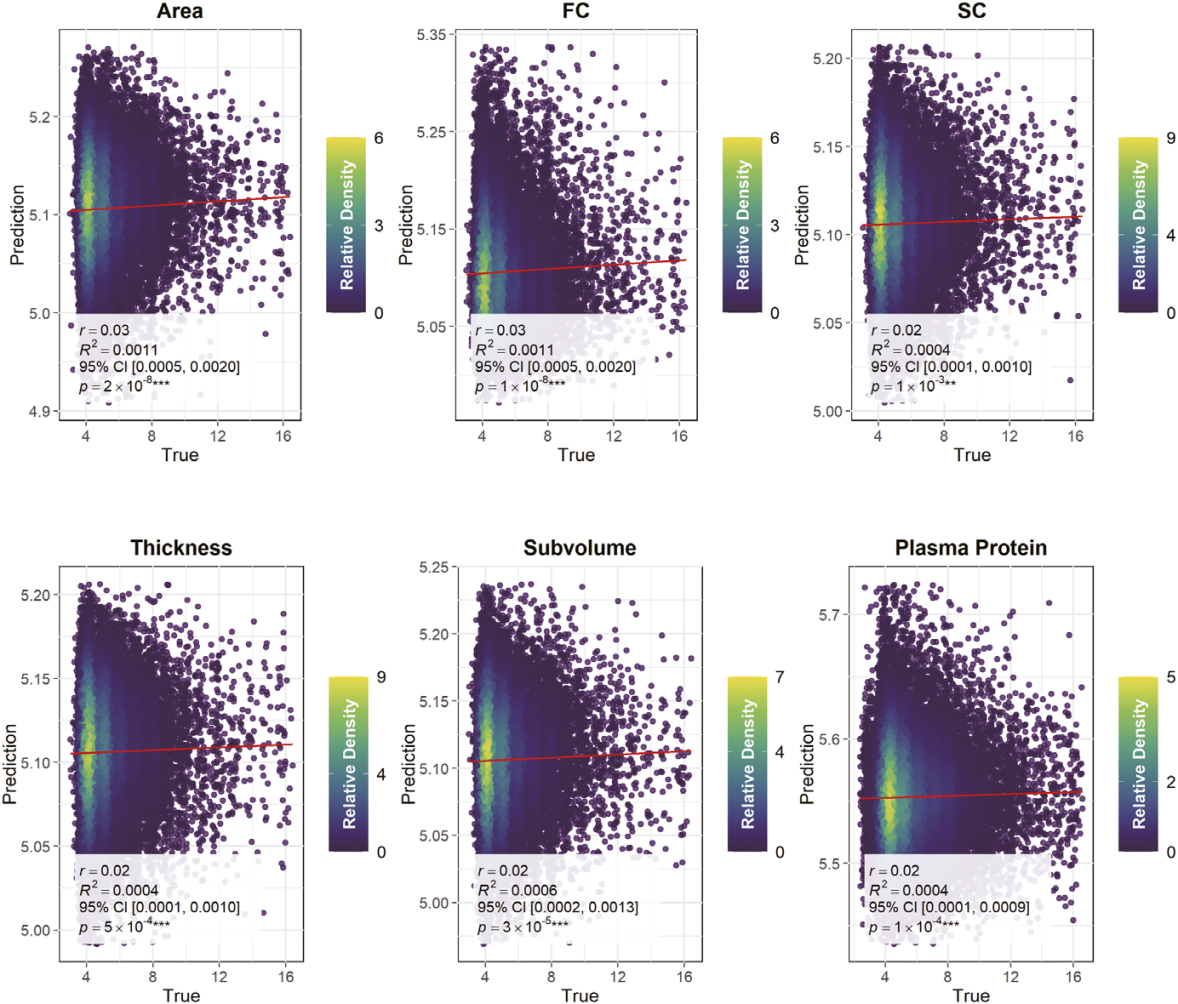
Association of RDS-4 depression symptom scores with NeuroPro-Dep in the validation dataset. *Note*: For the plasma protein modality (N = 36,991) and five brain modalities (N = 28,645), we identified independent UKB subsets not included in the multimodal fusion analysis. Association models were constructed using mean values of proteins or neuroimaging features with high absolute loading Z-scores from the spatial maps derived in the fusion analysis, as well as the reconstructed loadings (detailed in Methods). The scatterplots illustrate the relationship between the actual RDS-4 scores and the scores derived from these components. Statistical metrics for each modality include the Pearson correlation coefficient (r), coefficient of determination (R2), 95% CI for R2, and p-values. (* *p* < .05, ** *p* < .01, *** *p* < .001).

The depression scale we used was the RDS-4, a measure reflecting the current depressive state of participants. Its assessment timing aligns with the measurement time of the modalities and it exhibits a strong correlation with the PHQ-9 scale (Dutt et al., 2022). As shown in Figure 3, the Pearson correlation coefficients and p-values for the associative strength of each modality on RDS-4 are as follows: plasma proteins (r = .02, p = 1×10^−4^ ***), cortical thickness (r = .02, p = 4.8×10^−4^ ***), cortical surface area (r = .03, p = 2×10^−8^ ***), subcortical volume (r = .02, p = 2.5×10^−5^ ***), FC (r = .03, p = 1.3×10^−6^ ***), and SC (r = .02, p = 1.1×10^−3^ **). The predicted values from each modality-specific feature of NeuroPro-Dep were significantly correlated with the true RDS-4 scores, indicating that this multimodal neuroimaging–proteomic covariation component of depression (NeuroPro-Dep) has detectable associations not only with the distinction between healthy individuals and depression patients but also with the depressive symptom severity of participants.

Although the observed correlation magnitudes appear small, such effect sizes are typical for large-scale brain–behavior association studies. A recent Nature publication (Marek et al., 2022) demonstrated that smaller neuroimaging studies often report inflated correlations compared with the largest effects reproducibly observed in large samples. Consistent with this, similarly small but robust effect sizes have been repeatedly reported in other large-sample UK Biobank analyses examining brain–mental health associations (Li et al., 2024; Qi et al., 2022).

**Figure 4.**
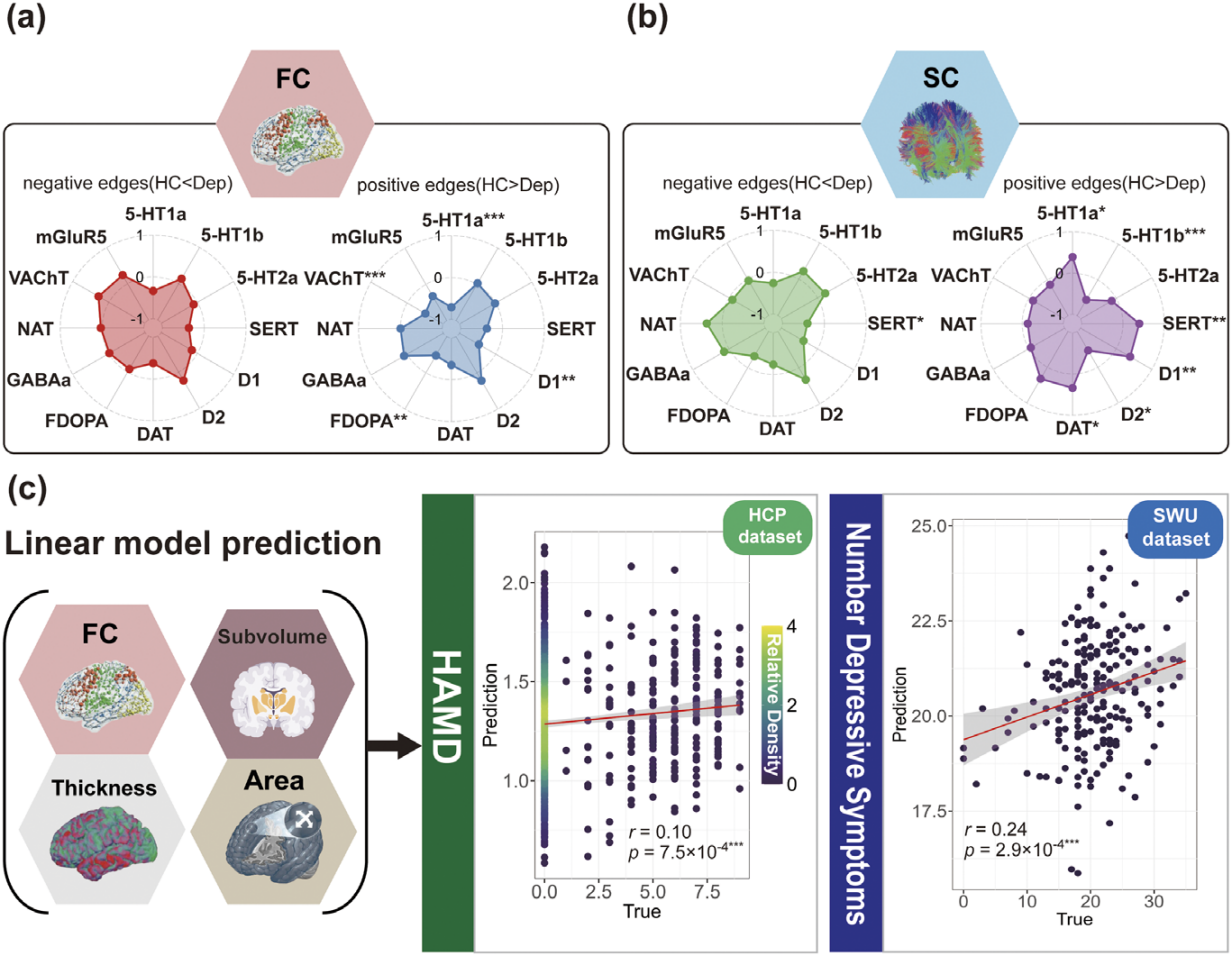
Associations between NeuroPro-Dep and neurotransmitters and its cross-dataset generalizability. *Note*: Panel (a) displays radar plots illustrating the associations between cumulative patterns of significantly negatively (red) and positively (blue) altered functional connectivity (FC) edges in healthy controls relative to depression patients and 12 neurotransmitter systems. Panel (b) shows similar radar plots for structural connectivity (SC), depicting associations with significantly negatively (green) and positively (purple) altered edges. Panel (c) examines the cross-dataset association strength of NeuroPro-Dep for depressive symptoms across two datasets. For the SWU Depression dataset, participants with diagnosed depression were selected, and a linear model was constructed using four neuroimaging modalities of NeuroPro-Dep to associate with HAMD scores. The Pearson correlation coefficient between predicted and actual scores is presented in the plot. For the HCP dataset, the number of depressive symptoms was used as the outcome measure, and the same process was applied to generate the result. (* *p* < .05, ** *p* < .01, *** *p* < .001).

We also conducted cross-sample validation (across external datasets involving diverse populations and multiple depression rating scales) to ensure the robustness and generalizability of the identified components. We applied the aforementioned methods to associate with two distinct depression scales using the HCP dataset (N = 1,084) and the SWU Depression dataset (N = 242), which exclusively includes individuals with depression. Since SWU Depression and HCP do not include plasma protein data – and UKB is one of the very few datasets simultaneously collecting neuroimaging and plasma protein data – we focused on assessing the combined association strength of the neuroimaging modalities of NeuroPro-Dep (see Methods for more details). In the SWU Depression dataset (Yan et al., 2019), depression severity was assessed using the Hamilton Depression Scale (HAMD), a well-established measure of depression severity. In the HCP dataset, the depression scale used was the number of depressive symptoms, consistent with previous studies in the literature (Yuan et al., 2022). As shown in Figure 4c, in the SWU Depression dataset, the neuroimaging modalities of NeuroPro-Dep significantly predicted HAMD scores in depression patients (r = 0.24, p = 2.9 × 10^−4^***). Similarly, in the HCP dataset, the neuroimaging modalities were significantly associated with the number of depressive symptoms (r = 0.10, p = 7.5 × 10^−4^***). These results demonstrate the generalizability of NeuroPro-Dep, indicating its significant association with depressive symptoms across different samples.

### Association of NeuroPro-Dep structural and functional connectivity patterns with neurotransmitter maps

Given that neurotransmitter dysregulation is widely considered a mechanism linking physical illness to depression, we posited that the connectivity alterations observed within the NeuroPro-Dep neuroimaging modalities may be related to changes in neurotransmitter receptor and transporter systems. To test this, we correlated the cumulative patterns of significantly altered edges (|Z| > 2)for each brain region in the spatial maps of SC and FC, with whole-brain maps of 12 neurotransmitter receptors and transporters using the JuSpace toolbox (Dukart et al., 2021) (Figure 4a,b).

As shown in Figure 4a,b, the analysis revealed several significant associations between neurotransmitter systems and the cumulative patterns of altered connectivity. For the cumulative patterns of significantly negatively altered FC edges (Figure 4a, red), significant associations were observed with the serotonin transporter (SERT, r = −0.37, p = .026*) and dopamine transporter (DAT, r = −0.38, p = .031*). In contrast, for the cumulative patterns of significantly positively altered FC edges (Figure 4a, blue), significant associations were identified with the serotonin receptor 5-HT1a (r = −0.71, p = 9.9 × 10^−4^***), dopamine receptor D1 (r = −0.46, p = .008**), dopamine precursor FDOPA (r = −0.48, p = .005**), and acetylcholine transporter VAChT (r = −0.50, p = 1 × 10^−3^***).

For the cumulative patterns of significantly negatively altered SC edges, significant associations were found with the serotonin transporter SERT (r = −0.40, p = .016*) (Figure 4b, green). Meanwhile, for the cumulative patterns of significantly positively altered SC edges (Figure 4b, purple), significant associations were observed with the serotonin 5-HT1a receptor (r = 0.39, p = .03*), the 5-HT1b serotonin receptor (r = −0.56, p = 1 × 10^−3^***), the SERT serotonin transporter (r = 0.40, p = .008**), the D1 dopamine receptor (r = 0.39, p = .038*), the D2 dopamine receptor (r = −0.47, p = .014*), the dopamine transporter DAT (r = 0.34, p = .046*), and the dopamine precursor FDOPA (r = −0.33, p = .029*). These findings suggest that the abnormal alterations in neuroimaging modalities of NeuroPro-Dep in depression patients may be influenced by neurotransmitter systems.

### Biological profiling and multilevel phenotypic associations of plasma proteins component in NeuroPro-Dep

After establishing the robustness of the NeuroPro-Dep multimodal neuroimaging–proteomic component, and having delineated the neurobiological correlates of its imaging features in the previous section, we next sought to characterize the biological mechanisms reflected specifically by its plasma protein component, given that plasma proteins reflect systemic physiological states. We first performed biological pathway and tissue-enrichment analyses to identify the molecular processes and organ systems represented by these proteins. Next, to provide a systems-level view of their coordinated regulation, we examined the distribution of these proteins across WGCNA co-expression modules. Building on these biological insights, we then characterized the broader phenotypic and genetic relevance of the NeuroPro-Dep plasma proteins signature.

**Figure 5.**
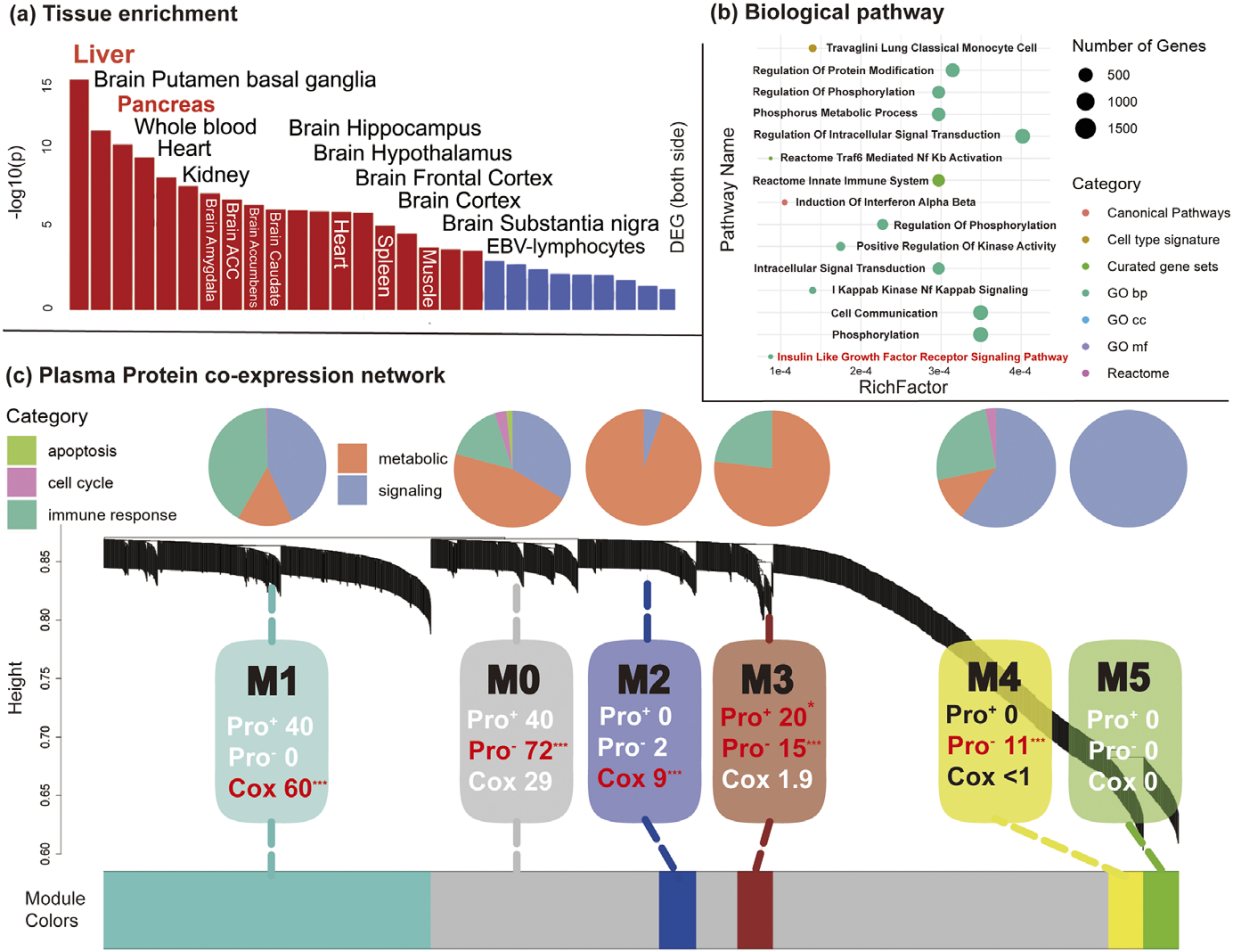
Biological pathways of plasma proteins in NeuroPro-Dep. *Note*: (a) FUMA gene functional annotation of genes corresponding to plasma proteins with |Z| > 3 in the multimodal neuroimaging–plasma protein covariation component of depression (NeuroPro-Dep) plasma protein modality, revealing tissue-specific expression. Significant tissues include the pancreas, liver, blood, and brain. (b) FUMA gene functional annotation of genes corresponding to plasma proteins with |Z| > 3, identifying enriched biological pathways. (c) Comparison of WGCNA module assignments between plasma proteins in NeuroPro-Dep (|Z| > 3) and proteins directly associated with depression diagnoses identified using the Cox regression model. *p*-values were calculated using hypergeometric distribution. The pie charts illustrate the proportion of biological process categories in each module, derived from GO enrichment analysis of all genes within the module. In the figure, pro^+^ represents the group of proteins from the NeuroPro-Dep plasma protein modality with Z>3, pro^-^ represents the group of proteins with Z<−3, and **cox** represents the group of proteins directly associated with depression diagnosis identified through the Cox model. The numbers represent the percentage of each module containing the specific group. (* *p* < .05, ** *p* < .01, *** *p* < .001).

To begin with, we carried out biological process and tissue-enrichment analyses. To identify the biological processes and organ systems represented by the NeuroPro-Dep plasma proteins, we first selected proteins with high positive or negative loadings (|Z| > 3) from the NeuroPro-Dep plasma protein spatial map. We then mapped these proteins to their corresponding genes, performed pathway and tissue-enrichment analyses using FUMA (Watanabe, Taskesen, van Bochoven, & Posthuma, 2017). Tissue specific expression analysis revealed that the genes corresponding to high loadings proteins in the plasma protein modality were significantly expressed in the pancreas, liver, blood, and brain (Figure 5a). Biological pathway enrichment analysis indicated that these proteins were primarily enriched in immune response-related pathways and involved in the insulin-like growth factor receptor signaling pathway, which is associated with metabolic regulation (Figure 5b).

Second, to investigate the coordinated expression patterns of the NeuroPro-Dep plasma proteins, we applied WGCNA to cluster proteins into co-expression modules. This approach provides a network-level perspective on how high loadings proteins (|Z| > 3) from the NeuroPro-Dep plasma proteins spatial map are organized in biological regulatory space. Moreover, to contextualize these modules and evaluate whether multimodal fusion identifies protein groups distinct from those discovered through univariate clinical association alone, we constructed a reference set of depression-related proteins using a Cox regression model examining the association with future depression incidence, following previous published work (Kang et al., 2025). We then compared the module distribution of NeuroPro-Dep proteins with this Cox-derived protein group. As shown in Figure 5c, NeuroPro-Dep positive high loadings proteins were predominantly enriched in Module 3, while negative proteins localized to Modules 0 and 3. In contrast, Cox-identified proteins aggregated mainly within Module 1, indicating that multimodal fusion captures a biologically distinct subset. Further pathway composition analysis revealed that Modules 0 and 3 – significantly containing NeuroPro-Dep proteins – were enriched for metabolic processes, whereas Module 1 – significantly containing Cox proteins – displayed more immune-related pathways.

**Figure 6.**
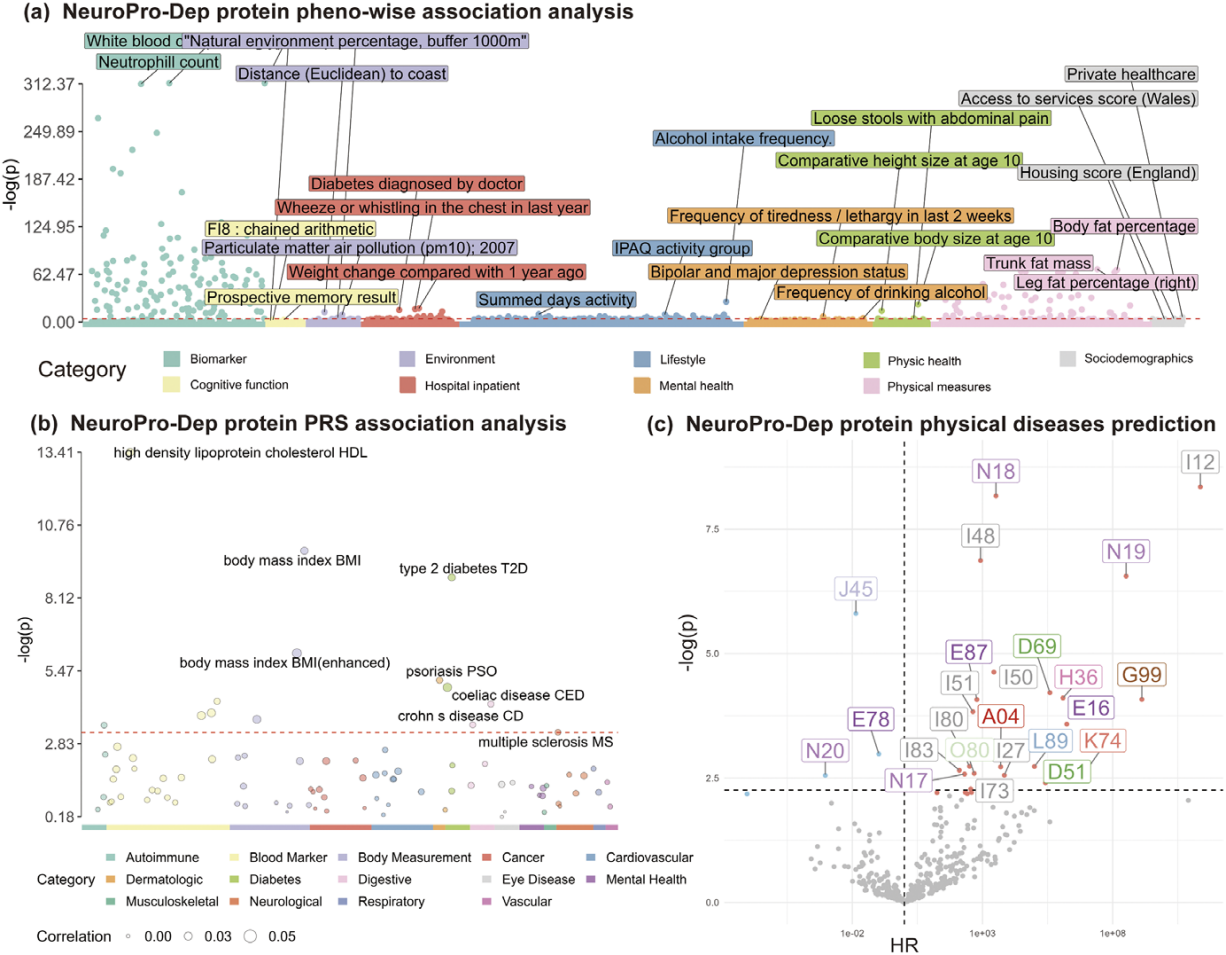
Association analyses of NeuroPro-Dep modalities with 1,200 multidimensional phenotypes and genetic risks. *Note*: (a) NeuroPro-Dep Protein-Phenotype Association Analysis: Using the protein modality of NeuroPro-Dep, a phenome-wide association analysis was conducted. The y-axis represents -log10(p-values), and phenotypes above the red line are significant after Bonferroni correction. Different colors represent different phenotype categories, with the top three significant phenotypes in each category labeled. (b) NeuroPro-Dep Protein-PRS Association Analysis: Using the protein modality of NeuroPro-Dep, association analyses were performed with polygenic risk scores (PRS) for physical diseases, measurements, and blood biomarkers. The y-axis represents -log10(p-values), and phenotypes above the red line are significant after Bonferroni correction. Different colors represent various phenotype categories, and the size of each point indicates the relative magnitude of the Pearson correlation coefficient (r) between predicted and actual values. (c) NeuroPro-Dep Protein-Physical Diseases association: Each point represents a physical disease identified by ICD-10 codes in the UKB dataset. The y-axis represents -log10(p-values), and the x-axis shows Hazard Ratios (HR). Diseases above the horizontal dashed line are significant after Bonferroni correction. Diseases to the left of the vertical dashed line (HR < 1) are shown in blue, indicating a protective influence of the plasma protein modality for the depression group. Diseases to the right (HR > 1) are shown in red, reflecting a risk-enhancing effect of the plasma protein modality for the depression group.

Finally, through the association analysis of NeuroPro-Dep plasma protein modality with comprehensive phenotypes, physical diseases onset, and polygenic risk scores for physical diseases, we identified a link between NeuroPro-Dep and metabolic diseases such as type 2 diabetes (p = 2.4 × 10^−21^***). Utilizing the Cox proportional hazards model, we demonstrate its robust association with the onset of various future physical diseases, including metabolic and cardiovascular disorders. These results are presented in Figure 6 and detailed interpretations can be found in Supplementary Materials’ eResults section.

### Mechanisms linking NeuroPro-Dep plasma proteins to depression

After demonstrating a strong association between the NeuroPro-Dep plasma protein and depression (Figure 3), and further observing that high loadings proteins were predominantly enriched in metabolic pathways (Figures 5 and 6), we next sought to determine whether these proteins play a causal role in depression through metabolic regulation. Motivated by the distinct metabolic signature linked to NeuroPro-Dep and by prior evidence connecting metabolic dysfunction with depression, we applied a two-step MR framework to evaluate whether plasma proteins causally influence depression risk via metabolic intermediates. Summary statistics for BMI, depression, and plasma protein GWAS were drawn from large-scale European cohorts and processed following standard instrument selection and harmonization procedures (see Methods).

**Figure 7.**
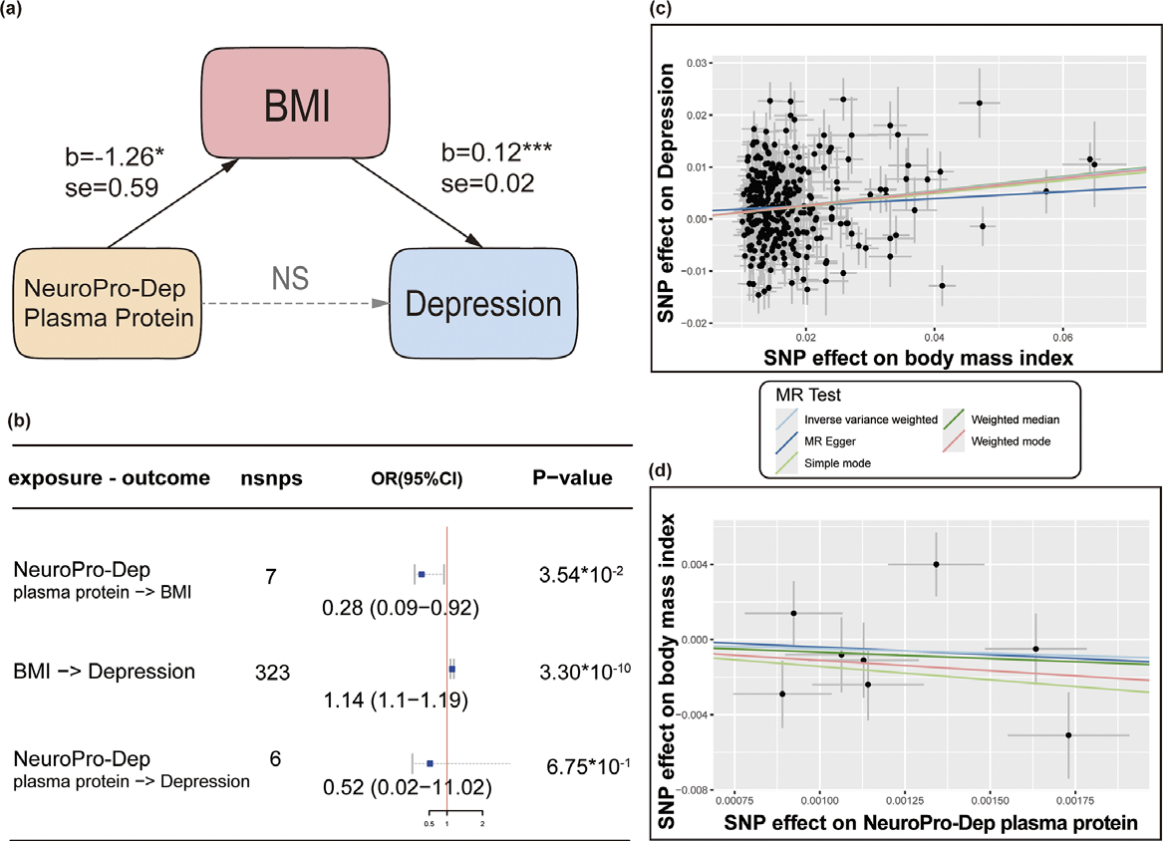
Two-step Mendelian randomization analysis reveals an indirect negative effect of NeuroPro-Dep plasma proteins on depression via BMI. *Note*: (a) Schematic representation of the indirect effect, where b represents the two-sample MR inverse variance weighted beta coefficient, se denotes the inverse variance weighted standard error, and NS indicates nonsignificant MR effects. (b) Table summarizing the MR effect estimates for proteins, BMI, and depression. Effect estimates represent the inverse variance weighted odds ratio for each outcome per one-point increment in the three exposures. Data are presented as mean values ± s.e.m. Nsnps refers to the number of instrument SNPs used in the analysis. The width of the lines extending from the center point represents the 95% confidence interval (CI). Two-sided unadjusted association *p*-values from Inverse variance weighted model are provided. (c) Scatterplot illustrating the SNP effects on BMI versus depression, with the slope of each line corresponding to the estimated MR effect for each method. Points represent raw β values for both variables, accompanied by 95% CI values. (d) Scatterplot showing SNP effects on NeuroPro-Dep plasma proteins versus BMI. Strongly associated IVs with NeuroPro-Dep plasma proteins, exhibiting independent genetic backgrounds, were selected.

As the first step, we assessed the causal relationship between NeuroPro-Dep protein levels and metabolic status. BMI was selected as a classic and crucial metabolic indicator to represent metabolic health, which was previously reported to have a unidirectional MR effect on depression (He et al., 2023)^,^(Berk et al., 2023). We chose a subgroup SNP strongly associated with NeuroPro-Dep plasma proteins (p < 1 × 10^−9^) as instrumental variable, and confirmed their genetic independence, as detailed in the Methods (see Figure 7a,b). We found a significant negative effect (OR = 0.28, p = .035), indicating that a decrease in NeuroPro-Dep plasma proteins leads to an increase in BMI. Sensitivity analyses were conducted and excluded the possibility of reverse causality (see Supplementary Tables S6–S9 and Supplementary Figure S3 for the full results). This aligns with the trend observed in Figure 1e, where depression patients exhibit lower NeuroPro-Dep plasma protein modality loadings levels.

Next, the two-sample MR analysis between BMI and depression (Figure 7a,b) revealed a significant positive effect was observed (OR = 1.14, p = 3.3 × 10^−10^), consistent with previous findings in the literature. Detailed sensitivity analysis is provided in Supplementary Tables S10–S13 and Supplementary Figure S4, which also rules out reverse causality. Finally, a direct two-sample MR analysis between NeuroPro-Dep plasma proteins and depression shown no significant effect (OR = 0.52, p = 0.67), as shown in Figure 7b, although the direction of the effect remained consistent with the MR to BMI. Taken together, these findings confirm an indirect causal effect of NeuroPro-Dep plasma proteins on depression through BMI.

## Discussion

In this study, we identified a multimodal covariation pattern, constrained by depression diagnoses, that integrates plasma proteins with brain structural and functional imaging features (including cortical thickness, cortical surface area, subcortical volume, FC, and SC). This pattern, termed multimodal neuroimaging–proteomic covariation component of depression (NeuroPro-Dep), provides insights into the biological pathways and bidirectional interactions underlying the comorbidity of depression and physical diseases. To the best of our knowledge, this is the first attempt to fuse plasma proteins, depression diagnoses, and brain structural and functional features into a unified framework. NeuroPro-Dep exhibits robust associations with depression symptoms across datasets and reveals the causal effect of plasma protein modalities on depression via metabolic dysfunction, offering new evidence for metabolic-based interventions.

In our study, NeuroPro-Dep incorporates multiple brain structural and functional features, revealing key cross-modal brain regions significantly associated with depression diagnoses and the plasma protein modality. These regions include the sensorimotor cortex, temporal lobe, subcallosal gyrus, and subcortical structures such as the thalamus and hippocampus, which are commonly reported in previous studies on depression-related brain imaging abnormalities (Otte et al., 2016). Our findings in the sensorimotor and prefrontal areas are particularly resonant with current large-scale evidence. Specifically, the alterations in primary regions mirror the transcriptomic-driven ‘hypo-function’ observed in recent spatial correlation analyses (Zhu et al., 2024). Moreover, the involvement of prefrontal structures aligns with evidence that the brain’s structural connectome constrains the spatial propagation of cortical atrophy, often centering on prefrontal hubs (Shen et al., 2025). Importantly, our results offer novel multimodal evidence implicating the subcallosal gyrus – a region previously identified as a critical target for depression treatment(Crowell et al., 2019; Hamani et al., 2011; Lozano et al., 2008), and reinforce prior findings of its abnormal metabolic activity in depression (Mayberg et al., 2005).

Interestingly, NeuroPro-Dep revealed a decreased cortical surface area in the left superior temporal sulcus and an increased surface area in the right, suggesting a potential morphological asymmetry that future studies could explore in relation to depression-related plasma protein profiles. This morphological asymmetry aligns with the established functional lateralization of the STS (Specht & Wigglesworth, 2018). Specifically, the left STS is primarily involved in the integration of language-related social cues and semantic processing (Kausel, Michon, Soto-Icaza, & Aboitiz, 2024; Özyürek, 2014). A reduction in its surface area may therefore correspond to impaired processing of positive social-semantic information in depression, a notion supported by widespread temporal abnormalities observed in large-scale MDD cohorts (Kochunov et al., 2025; Schmaal et al., 2017). Conversely, the right STS is consistently linked to socio-emotional decoding and emotion recognition (Alaerts et al., 2014). The observed relative enlargement of the right STS might reflect a compensatory structural adaptation to the sustained negative emotional load characteristic of depressive states, although such a mechanism warrants further direct longitudinal validation.

The NeuroPro-Dep revealed a series of proteins with significantly altered expression in depression, as identified through fusion with brain structure and function data, particularly those with upregulated levels. Among these, IRAG2, TBC1D5, PTPN1, MAPK35, DNMBP, NMNAT1, and CRACR2A were notable proteins, involved, respectively, in glucose metabolism (IRAG2; Prüschenk, Majer, & Schlossmann, 2023), intracellular transport and neurodegeneration (TBC1D5; Liang et al., 2024), insulin signaling (PTPN1; Gurzov, Stanley, Brodnicki, & Thomas, 2015), stress response (MAPK35; Haim et al., 2017), Alzheimer’s disease risk (DNMBP; Bettens et al., 2009), neuroprotection (NMNAT1; Sasaki et al., 2016), and immune regulation (CRACR2A; Wang et al., 2019). Conversely, significantly downregulated proteins included GH1, reduced in obesity (Vakili, Jin, & Cattini, 2014), and IGFBP1, linked to insulin resistance and cardiometabolic risk (Rajwani et al., 2012). In summary, the proteins identified in this study are predominantly involved in metabolic processes, alongside pathways related to neuroprotection, neurodegeneration, and immune regulation.

Considering its covarying pattern with brain imaging features, we aim to investigate whether the plasma protein modality of NeuroPro-Dep differs from previous findings that directly associated depression phenotypes with plasma proteins. To do this, we compared our results with a study that also utilized UKB data to explore the relationship between plasma proteins and depression (Kang et al., 2025). The prior study identified key proteins such as GDF-15, LRRN1, TNF, and BTNA32, which were primarily linked to immune inflammation, neuroprotection, and neurodegenerative diseases. In contrast, our study highlighted proteins including IRAG2, PTPN1, DNMBP, NMNAT1, CRACR2A, and GH1, which are predominantly associated with metabolic dysregulation, neuroprotection, neurodegenerative diseases, and immune processes. Notably, our findings emphasize the role of metabolic-related proteins, distinguishing our results from the previous research. In contrast to previous studies that have investigated the etiology of depression in the UK Biobank (UKB) cohort using specific phenotypes – such as bodily pain (Jiang et al., 2025), physical frailty (Jiang et al., 2024), or healthy lifestyle (Y. Zhao et al., 2023) – our findings emphasize the critical role of metabolic dysregulation in the development of depression and its comorbidity with physical diseases, beyond the commonly implicated inflammatory pathways. Moreover, compared to phenotype-based markers, plasma proteins offer more direct targets for intervention and diagnosis, thereby providing valuable insights for the prevention and treatment of depression and its physical comorbidities.

One of the key findings revealed by the plasma protein modality of NeuroPro-Dep is the critical role of metabolic system-related pathways. First, tissue enrichment analysis of the plasma protein modality identified significant enrichment in the pancreas and liver. Second, phenotype association analysis showed significant associations between the plasma protein modality and insulin use as well as diabetes diagnoses. Additionally, the plasma protein modality is associated with future risks of ICD-10-coded physical diseases such as E87 (other disorders of fluid, electrolyte, and acid-base balance) and E16 (other disorders of pancreatic endocrine function). More importantly, the plasma protein modality was significantly associated with polygenic risk scores for type 2 diabetes, BMI, and HDL cholesterol.

Most notably, by adopting a two-step MR analysis, we identified a causal pathway through which NeuroPro-Dep plasma proteins influence depression via BMI. This brings the question of how metabolic system abnormalities, represented by BMI, may impact depression through brain functions. To begin with, the most important hormone in carbohydrate metabolism is insulin, and its dysregulation, which can lead to diabetes, has been shown to be associated with the comorbidity between type 2 diabetes and various mental disorders, particularly depression. A series of important studies has highlighted the critical role of brain insulin pathways in regulating metabolism, emotion, cognition, and behavior (Brüning et al., 2000). Insulin is thought to modulate the brain via insulin receptors and IGF-1 receptors, and among the key proteins identified in our study is IGFBP1, a regulatory protein for IGF-1. Additionally, insulin may influence the brain by regulating neurotransmitters. For example, studies have shown that insulin signaling in astrocytes modulates dopamine release through ATP release, which in turn affects depressive and anxious behavioral phenotypes (W. Cai et al., 2018). Moreover, insulin is known to regulate serotonin by directly modulating the activity of dorsal raphe 5-HT neurons, dampening 5-HT neurotransmission via a 5-HT1A receptor-mediated inhibitory feedback mechanism (Martin et al., 2024). Consistent with these findings, our results revealed that the structural connectivity (SC) and FC patterns identified in the NeuroPro-Dep brain modality were significantly associated with whole-brain maps of dopamine receptors and 5-HT1A receptors, suggesting a link between these neurotransmitter systems and the metabolic pathways mediated by insulin. In the future, a deeper understanding of brain IGF-1 and insulin pathways (Chen, Cai, Hoover, & Kahn, 2022), along with the investigation of regulatory strategies for the identified key plasma proteins – such as PTPN1 inhibitors (Cheyssac et al., 2006) – could provide promising avenues for treating depression by improving metabolic system function. Specifically, PTPN1 (also known as PTP1B) serves as a classic negative regulator of both insulin and leptin signaling pathways, positioning it as a pivotal node in the brain–body metabolic axis (Delibegović, Dall’Angelo, & Dekeryte, 2024; Zhang, Dodd, & Tiganis, 2015). The therapeutic potential of PTPN1 lies in its ability to restore metabolic sensitivity; however, its application as an antidepressant requires overcoming challenges such as enhancing blood–brain barrier permeability and mitigating systemic side effects (Delibegović et al., 2024). Complementing these extracellular metabolic signals, IRAG2 acts as a crucial intracellular mediator by regulating calcium release via the 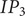 receptor, thereby linking hormonal inputs to downstream neuronal and endocrine responses (Okumura et al., 2022; Prüschenk et al., 2023). While the translational feasibility of IRAG2 is currently contingent on the development of specific small-molecule modulators to minimize pleiotropic effects, its central role in coordinating cellular signaling kinetics underscores its value as a novel candidate for targeted intervention.

This study offers several advantages over previous research. Adopting a holistic approach to the interplay between the brain and body, we identified NeuroPro-Dep, a multimodal covariation pattern integrating brain structural and functional features, plasma proteins, and depression diagnoses. To the best of our knowledge, this represents the first study of its kind, offering a novel research paradigm for understanding the interaction between depression and physical diseases. Furthermore, NeuroPro-Dep was shown to significantly associate with depression symptom scores across datasets, further validating its credibility as a potential multimodal multimodal biological signature and therapeutic target for depression. Most importantly, we discovered that the plasma protein modality of NeuroPro-Dep influences depression through BMI, revealing causal mechanisms and providing deeper insights into the metabolic intervention perspective for depression and the prevention of secondary physical diseases.

Several limitations of this study warrant consideration. First, the UKB cohort primarily consists of individuals of European ancestry, limiting the cross-ethnic generalizability of NeuroPro-Dep. Second, the discovery phase involved a significant case-control imbalance (360 depression cases vs. >3,600 controls), which could potentially influence the stability of the high-dimensional multimodal fusion. To partially mitigate this concern, we performed a rigorous downsampling sensitivity analysis using a balanced 1:1 case-control ratio over 50 iterations (Supplementary Figure S9), which revealed robust spatial stability for the core biological features of NeuroPro-Dep Third, while we performed cross-dataset validation, the available external cohorts (e.g. HCP and SWU) lack plasma proteomics modality. Because the UK Biobank is currently one of the few large-scale datasets that simultaneously collects both high-dimensional neuroimaging and proteomics, our external validation was necessarily restricted to the imaging-derived components of the model. Future studies leveraging expanded datasets or new large-scale cohorts with dual-modality data will be essential to further validate the full multimodal framework and its applicability across more diverse populations. Finally, the use of diverse psychometric constructs across datasets (e.g. RDS-4, HAMD, and symptom counts) introduces inherent measurement heterogeneity. However, prior work suggests that these depression-related measures are substantially interrelated and capture overlapping core features of depressive symptomatology (Dutt et al., 2022; Reilly, MacGillivray, Reid, & Cameron, 2015), such that the consistent performance of NeuroPro-Dep across them may indicate sensitivity to a common biological substrate rather than to scale-specific item content.

## Conclusion

In conclusion, this study identified NeuroPro-Dep, a multimodal covariation component comprising plasma proteins, brain structural and functional features, and depression diagnoses, to elucidate the interaction between depression and physical diseases. The robust cross-dataset associative patterns of NeuroPro-Dep further underscore its potential as a multimodal biological signature for depression. Notably, causal analysis of the plasma protein modality in NeuroPro-Dep, which covaries with brain structure and function patterns, uncovered mechanisms by which metabolism influences depression. This finding provides new insights into potential intervention targeting the metabolic system for depression, highlights the importance of preventing secondary physical diseases associated with depression, and underscores the value of multimodal neuroimaging-plasma protein fusion in uncovering novel psychiatric biological signature from a body–brain interacting perspective.

## Supporting information

10.1017/S003329172610436X.sm001 Lian et al. supplementary material Lian et al. supplementary material

## Data Availability

The UK Biobank data are available on application at https://www.ukbiobank.ac.uk/.

## Abbreviations

BMI: body mass index
DAN: dorsal attention network
DMN: default mode network
FC: functional connectivity
FPN: frontoparietal network
GWAS: Genome-Wide Association Study
HAMD: Hamilton Depression Scale
HCP dataset: Human Connectome Project dataset
HDL: High-Density Lipoprotein
HPA: Hypothalamic-Pituitary-Adrenal
HR: Hazard Ratios
ICD-10: International Classification of Diseases, 10th Revision
LIM: limbic network
MCCAR+JICA: multi-site canonical correlation analysis with reference plus joint independent component analysis
MR: Mendelian randomization
MRI: Magnetic Resonance Imaging
NeuroPro-Dep: multimodal neuroimaging-plasma protein component of depression
OR: Odds Ratio
PHQ-9: Patient Health Questionnaire-9
PRS: Polygenic Risk Score
RDS-4: Recent Depressive Symptoms – 4 items
SC: structural connectivity
SNP: Single Nucleotide Polymorphism
SOM: sensorimotor network
SUB: subcortical network
Subvolume: subcortical volume
SWU dataset: Southwestern UniveristyUniversity (China) depression dataset
UKB: UKBiobank
VAN: ventral attention network
VIS: visual network
WGCNA: Weighted Gene Co-expression Network Analysis

## Supplementary material

The supplementary material for this article can be found at http://doi.org/10.1017/S003329172610436X.
