# Supplementary material for: Multimodal fusion of brain imaging and proteomics reveals a brain–body pathway linking depression and metabolic dysfunction
Source: Psychol Med. 2026 May 13;56:e144. doi: 10.1017/S003329172610436X (PMC13200150; doi:10.1017/S003329172610436X)
Supplement: Lian et al. supplementary material [file S003329172610436Xsup001.docx]

**Supplementary Materials**

**eTable**

eTable 1: Demographic Information of the UKB Discovery Cohort

|  | **depression** | **health** |
| --- | --- | --- |
|  | ***N=360*** | ***N=3606*** |
| Sex_0_0: |  |  |
| female | 253 (70.3%) | 1934 (53.6%) |
| male | 107 (29.7%) | 1672 (46.4%) |
| AgeAttend_0_0 | 52.0 [47.0;59.0] | 55.0 [48.0;61.0] |
| AgeAttend_2_0 | 61.0 [55.0;68.0] | 63.0 [57.0;69.0] |
| Ethnic_0_0: |  |  |
| Asian or Asian British | 22 (6.11%) | 115 (3.19%) |
| Black or Black British | 1 (0.28%) | 14 (0.39%) |
| Chinese | 0 (0.00%) | 6 (0.17%) |
| Mixed | 6 (1.67%) | 126 (3.49%) |
| Other | 1 (0.28%) | 24 (0.67%) |
| White | 330 (91.7%) | 3321 (92.1%) |
|  |  |  |

eTable 2：Demographic Information of the UKB Brain Imaging Validation Cohort

|  | **depression** | **health** |
| --- | --- | --- |
|  | ***N=2530*** | ***N=26115*** |
| Sex_0_0: |  |  |
| female | 1714 (67.7%) | 13937 (53.4%) |
| male | 816 (32.3%) | 12178 (46.6%) |
| AgeAttend_2_0 | 63.0 [57.0;69.0] | 65.0 [58.0;70.0] |
| Ethnic_0_0: |  |  |
| Asian or Asian British | 90 (3.56%) | 846 (3.24%) |
| Black or Black British | 5 (0.20%) | 85 (0.33%) |
| Chinese | 3 (0.12%) | 90 (0.34%) |
| Mixed | 63 (2.49%) | 778 (2.98%) |
| Other | 18 (0.71%) | 145 (0.56%) |
| White | 2351 (92.9%) | 24163 (92.6%) |
|  |  |  |

eTable3：Demographic Information of the UKB Plasma Protein Validation Cohort

|  | **depression** | **health** |
| --- | --- | --- |
|  | ***N=2782*** | ***N=34027*** |
| Sex_0_0: |  |  |
| female | 1854 (66.6%) | 18400 (54.1%) |
| male | 928 (33.4%) | 15627 (45.9%) |
| AgeAttend_0_0 | 57.0 [50.0;62.0] | 59.0 [51.0;64.0] |
| Ethnic_0_0: |  |  |
| Asian or Asian British | 85 (3.06%) | 1207 (3.55%) |
| Black or Black British | 21 (0.75%) | 210 (0.62%) |
| Chinese | 0 (0.00%) | 124 (0.36%) |
| Mixed | 89 (3.20%) | 1568 (4.61%) |
| Other | 34 (1.22%) | 425 (1.25%) |
| White | 2553 (91.8%) | 30493 (89.6%) |
|  |  |  |

eTable4：Demographic Information of the HCP Validation Cohort

|  | **HCP** | **N** |
| --- | --- | --- |
|  | ***N=1,084*** |  |
| Gender: |  |  |
| female | 587 (54.2%) | 1084 |
| male | 497 (45.8%) |  |
| Age | 28.8 (3.70) | 1084 |
| Race: |  |  |
| Am. Indian/Alaskan Nat. | 2 (0.18%) | 1084 |
| Asian/Nat. Hawaiian/Othr Pacific Is. | 65 (6.00%) |  |
| Black or African Am. | 158 (14.6%) |  |
| More than one | 29 (2.68%) |  |
| Unknown or Not Reported | 18 (1.66%) |  |
| White | 812 (74.9%) |  |
|  |  |  |

eTable5：Demographic Information of the SWU depression Validation Cohort

|  | **SWU** | **N** |
| --- | --- | --- |
|  | ***N=242*** |  |
| Gender: |  |  |
| female | 157 (64.9%) | 242 |
| male | 85 (35.1%) |  |
| Age | 38.7 (13.5) | 242 |
| ethnic: Chinese | 242 (100%) | 242 |
|  |  |  |

eTable6：NeuroPro-Dep plasma protein->BMI mr results

| outcome | exposure | method | nsnp | b | se | pval |
| --- | --- | --- | --- | --- | --- | --- |
| body mass index \|\| id:ieu-b-40 | NeuroPro-Dep plasma protein | MR Egger | 7 | -2.37 | 2.6019289 | 0.40442414 |
| body mass index \|\| id:ieu-b-40 | NeuroPro-Dep plasma protein | Weighted median | 7 | -0.90 | 0.7764399 | 0.24525910 |
| body mass index \|\| id:ieu-b-40 | NeuroPro-Dep plasma protein | Inverse variance weighted | 7 | -1.26 | 0.5968308 | .03535237 |
| body mass index \|\| id:ieu-b-40 | NeuroPro-Dep plasma protein | Simple mode | 7 | -1.16 | 1.3033891 | 0.40886006 |
| body mass index \|\| id:ieu-b-40 | NeuroPro-Dep plasma protein | Weighted mode | 7 | -0.82 | 1.2346259 | 0.53243163 |

eTable7：NeuroPro-Dep plasma protein->BMI pleiotropy test

| outcome | exposure | egger_intercept | se | pval |
| --- | --- | --- | --- | --- |
| body mass index \|\| id:ieu-b-40 | NeuroPro-Dep plasma protein | .0014 | .003164672 | 0.6776669 |

eTable8：NeuroPro-Dep plasma protein-> BMI heterogeneity test

| outcome | exposure | method | Q | Q_df | Q_pval |
| --- | --- | --- | --- | --- | --- |
| body mass index \|\| id:ieu-b-40 | NeuroPro-Dep plasma protein | MR Egger | 5.669484 | 5 | 0.3397189 |
| body mass index \|\| id:ieu-b-40 | NeuroPro-Dep plasma protein | Inverse variance weighted | 5.889949 | 6 | 0.4356304 |

eTable9:NeuroPro-Dep plasma protein->BMI steiger test

| outcome | exposure | snp_r2.exposure | snp_r2.outcome | correct_causal_direction | steiger_pval |
| --- | --- | --- | --- | --- | --- |
| body mass index \|\| id:ieu-b-40 | NeuroPro-Dep plasma protein | .01163918 | .00001539925 | TRUE | 3.66*10^-85^ |

eTable10：bmi->depression mr results

| outcome | exposure | method | nsnp | b | se | pval |
| --- | --- | --- | --- | --- | --- | --- |
| depression | body mass index \|\| id:ieu-b-40 | MR Egger | 348 | .068 | .05595932 | 0.23 |
| depression | body mass index \|\| id:ieu-b-40 | Weighted median | 348 | 0.14 | .02211916 | 7.2*10^-10^ |
| depression | body mass index \|\| id:ieu-b-40 | Inverse variance weighted | 348 | 0.14 | .02052498 | 4.37*10^-11^ |
| depression | body mass index \|\| id:ieu-b-40 | Simple mode | 348 | 0.15 | .07791503 | .053 |
| depression | body mass index \|\| id:ieu-b-40 | Weighted mode | 348 | 0.14 | .03915525 | 5.00*10^-4^ |

eTable11：bmi->depression pleiotropy test

| outcome | exposure | egger_intercept | se | | pval |
| --- | --- | --- | --- | --- | --- |
| depression | body mass index \|\| id:ieu-b-40 | .00125321 | | .0009682238 | 0.20 |

eTable12: bmi->depression heterogeneity test

| outcome | exposure | method | Q | Q_df | Q_pval |
| --- | --- | --- | --- | --- | --- |
| depression | body mass index \|\| id:ieu-b-40 | MR Egger | 1,211.616 | 346 | 1.90*10^-96^ |
| depression | body mass index \|\| id:ieu-b-40 | Inverse variance weighted | 1,217.482 | 347 | 4.34*10^-97^ |

Although some degree of heterogeneity among instrumental variables was observed in the two-sample MR analysis from BMI to depression, we employed the random-effects inverse variance weighted (IVW-MRE) model, which corresponds to the IVW results mentioned earlier, in the primary analysis. This model effectively mitigates the impact of heterogeneity on causal estimates by incorporating a random-effects term, thereby yielding more conservative and robust results. Sensitivity analyses indicated that the core conclusions remained consistent with results from alternative methods, such as MR-Egger and weighted median, further reinforcing the reliability of the findings. Additionally, previous MR studies in the literature also support a unidirectional causal relationship from BMI to depression(He et al., 2023).

eTable13: bmi->depression steiger test

| outcome | exposure | snp_r2.exposure | snp_r2.outcome | correct_causal_direction | steiger_pval |
| --- | --- | --- | --- | --- | --- |
| depression | body mass index \|\| id:ieu-b-40 | .03935477 | .001028899 | TRUE | 0 |

eTable14: NeuroPro-Dep plasma protein->depression mr results

| outcome | exposure | method | nsnp | b | se | pval |
| --- | --- | --- | --- | --- | --- | --- |
| depression | NeuroPro-Dep plasma protein | MR Egger | 6 | -10.33 | 9.870049 | 0.3543999 |
| depression | NeuroPro-Dep plasma protein | Weighted median | 6 | -0.18 | 1.487021 | 0.9040503 |
| depression | NeuroPro-Dep plasma protein | Inverse variance weighted | 6 | -0.65 | 1.557318 | 0.6751859 |
| depression | NeuroPro-Dep plasma protein | Simple mode | 6 | 0.44 | 2.785788 | 0.8799283 |
| depression | NeuroPro-Dep plasma protein | Weighted mode | 6 | -0.18 | 2.468539 | 0.9452058 |

**eFigure**

**
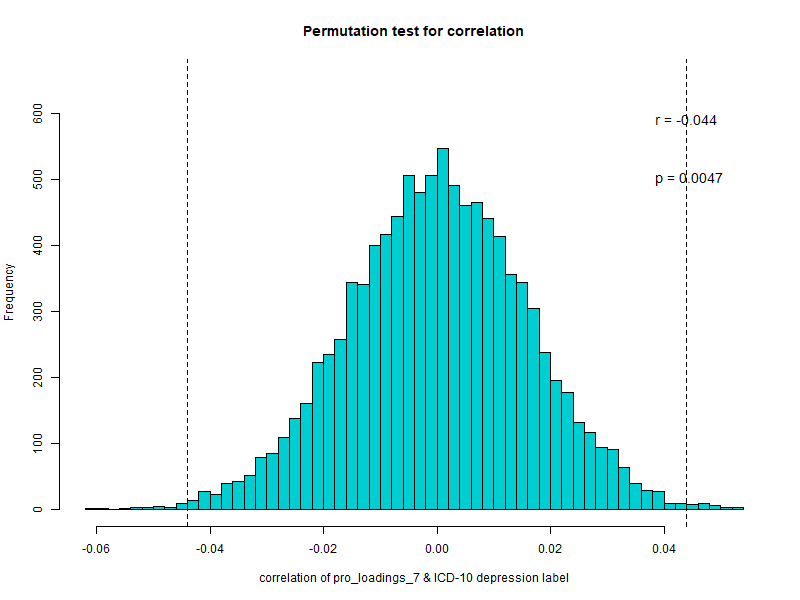
**

eFigure 1: Permutation tests for the correlations between NeuroPro-Dep modality loadings and reference signal（ICD-10 depression status）

To further validate the robustness of the multimodal associations identified by NeuroPro-Dep, we performed a non-parametric permutation test for each modality, following a procedure similar to that described in previous study(Sui et al., 2018). Specifically, the diagnostic labels (Y) were randomly permuted across participants 10,000 times, while keeping the modality-specific loadings ($A_{k}$) fixed. For each permutation, the correlation between $A_{k}$ and Y was recalculated to generate an empirical null distribution of r values under the assumption of no association. The empirical p value was computed as the proportion of permuted r values exceeding the observed correlation in magnitude.

As illustrated in eFigure 1, the permutation test for the plasma-protein modality is shown as a representative example. The empirical null distribution of correlation coefficients was approximately symmetric around zero, confirming that the permutation procedure effectively disrupted the true associations in the data. The observed correlation for this modality (r = −0.044, p = 0.0047) clearly deviated from the null distribution. Consistent with this example, all other modalities exhibited correlations lying in the extreme tails of their respective null distributions, with two-tailed permutation p values remaining significant after FDR correction (area = 0.0482, FC = 0.0433, plasma protein = 0.0141, SC = 0.0308, thickness = 0.0176, volume = 0.0084).

Together, these permutation-based significance estimates confirm that the observed cross-modal associations between the NeuroPro-Dep loadings and depression diagnosis are highly unlikely to have occurred by chance. This finding indicates that NeuroPro-Dep captures a meaningful multimodal pattern rather than a random noise component. Importantly, the same permutation procedure was consistently applied across all modalities to ensure methodological coherence with the validation and association analyses described in the main text.

eFigure 2: Scatter plot of the two-sample Mendelian Randomization (MR) analysis between NeuroPro-Dep plasma protein modal and depression.

As shown in eFigure 2 and Table 14, although the two-sample MR analysis from NeuroPro-Dep plasma protein modality to depression was not statistically significant, the directionality of the effect aligns with the pathway from plasma protein to body mass index (BMI) and subsequently to depression.

eFigure 3: Leave-one-out plot of the two-sample Mendelian Randomization (MR) analysis from BMI to depression The red line represents the causal effect estimate from the full model, while the black line indicates the estimates and confidence intervals after the exclusion of each individual SNP. After removing SNPs rs12125515 and rs1354034, the results remained significant. Exclusion of the other SNPs led to confidence intervals crossing zero, suggesting that the current significance partially depends on the contribution of these variables, though the effect direction remained consistent across all analyses. Given that the significance and direction of the primary analysis (IVW-MRE) were not overly influenced by any single SNP, and various sensitivity methods support a negative association, the conclusions appear to be relatively robust. However, this dependence on certain SNPs warrants caution in interpreting the results, and future large-sample validation may address this issue.


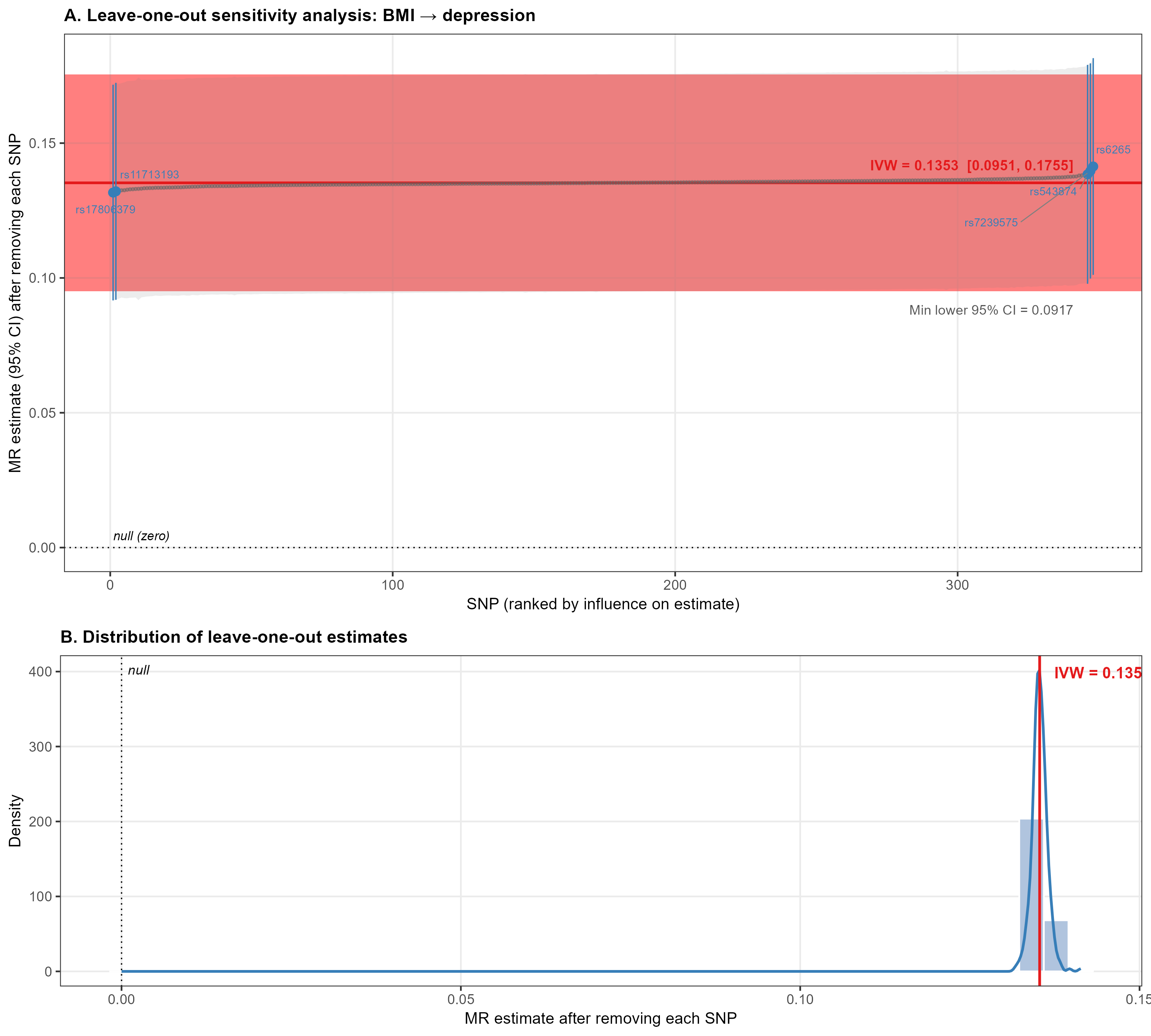


eFigure 4: Leave-one-out plot of the two-sample Mendelian Randomization (MR) analysis from BMI to depression


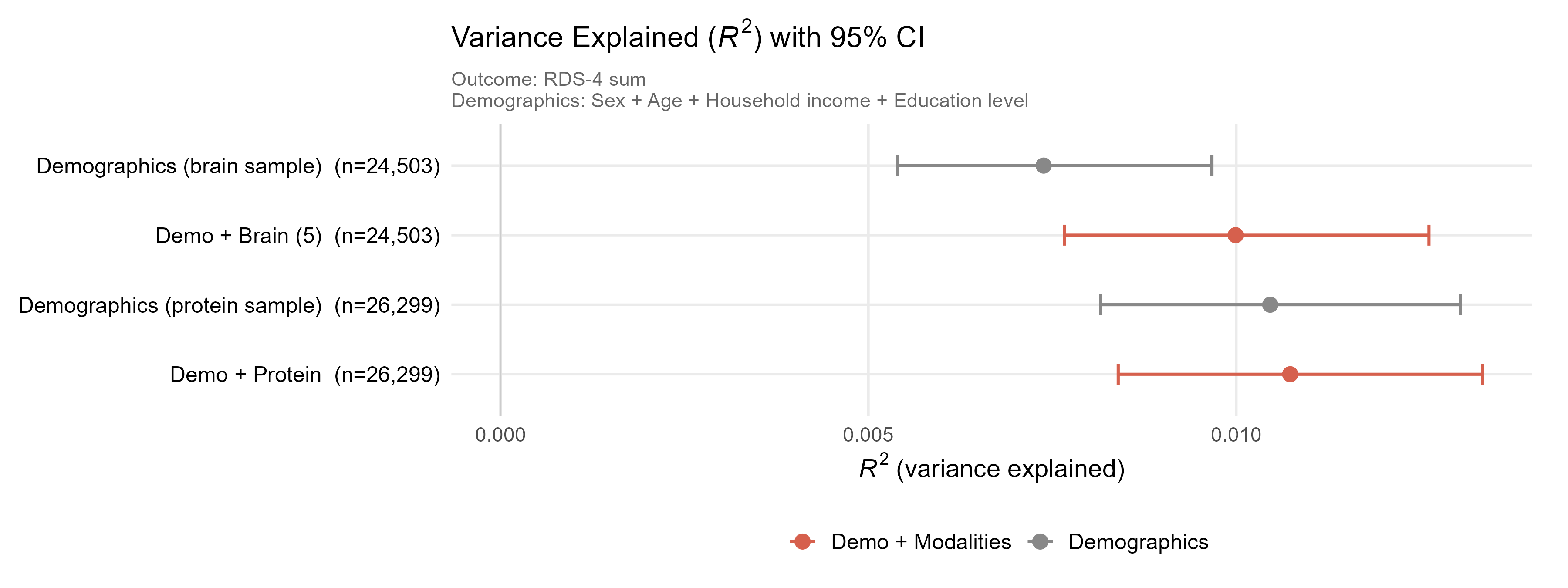


eFigure 5: Quantifying the predictive contribution of NeuroPro-Dep modalities. The forest plot displays the variance explained (R²) and its corresponding 95% confidence intervals (CI) for predicting depressive symptom severity (RDS-4 sum). To ensure methodological rigor, the neuroimaging sample (n = 24,503) and the proteomics sample (n = 26,299) were analyzed separately due to their non-overlapping participant distributions in the UK Biobank. Demographic Baseline (Grey): Includes age, sex, household income, and education level. Integrated Models (Orange): Represent the demographic baseline combined with either the five neuroimaging modalities (Demo + Brain (5)) or the plasma protein modality (Demo + Protein).

In response to concerns regarding the practical significance of the identified associations, we quantified the incremental value of the NeuroPro-Dep modalities over traditional demographic predictors (age, sex, income, and education). Given the non-overlapping nature of the imaging and proteomic validation cohorts in the UK Biobank, we established separate demographic baselines for each sample to provide a fair and accurate comparison.

Our results demonstrate that the addition of multimodal brain features and plasma protein profiles consistently increases the variance explained (R²) for depressive symptoms beyond what is captured by demographic factors alone. For the neuroimaging cohort, the integrated model (Demo + Brain (5)) showed a detectable increase in R² compared to the demographic-only model. In the proteomics cohort, the integrated model (Demo + Protein) also exhibited an incremental shift, though the gain was modest. This is partially expected given that the proteomic and neuroimaging data were acquired from non-overlapping UKB validation cohorts, limiting the multimodal synergy observed in the brain sample.


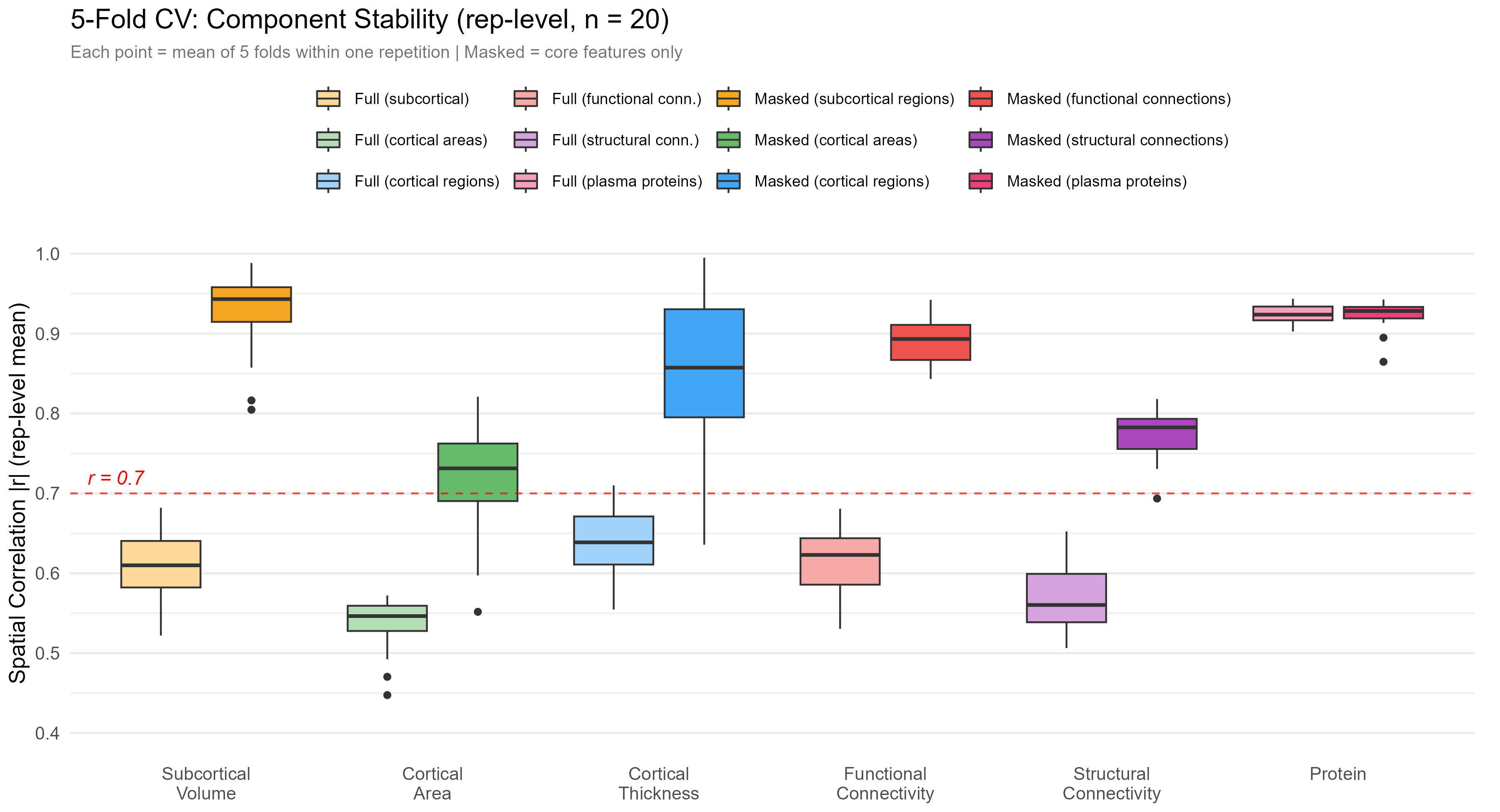


eFigure 6: Robustness of multimodal feature extraction across randomized sub-samples. To evaluate the stability of the identified NeuroPro-Dep component (Original Component 7), we performed a repeated 5-fold stratified cross-validation (20 repetitions x 5 folds = 100 iterations). In each iteration, the entire MCCAR+jICA fusion pipeline was re-executed using the training set (80% of the discovery cohort), and the resulting components were compared with the original NeuroPro-Dep spatial maps across six modalities. Each boxplot summarizes 20 repetition-level values, where each value is the mean spatial correlation |r| across the 5 folds within one repetition. Light-colored bars indicate correlations using the full feature vectors, while dark-colored bars represent "masked" correlations focusing exclusively on core features (i.e., brain features/proteins with high absolute spatial Z-scores in the original component as shown in main text figure 2). The dashed red line at |r| = 0.7 serves as a benchmark for high spatial consistency.


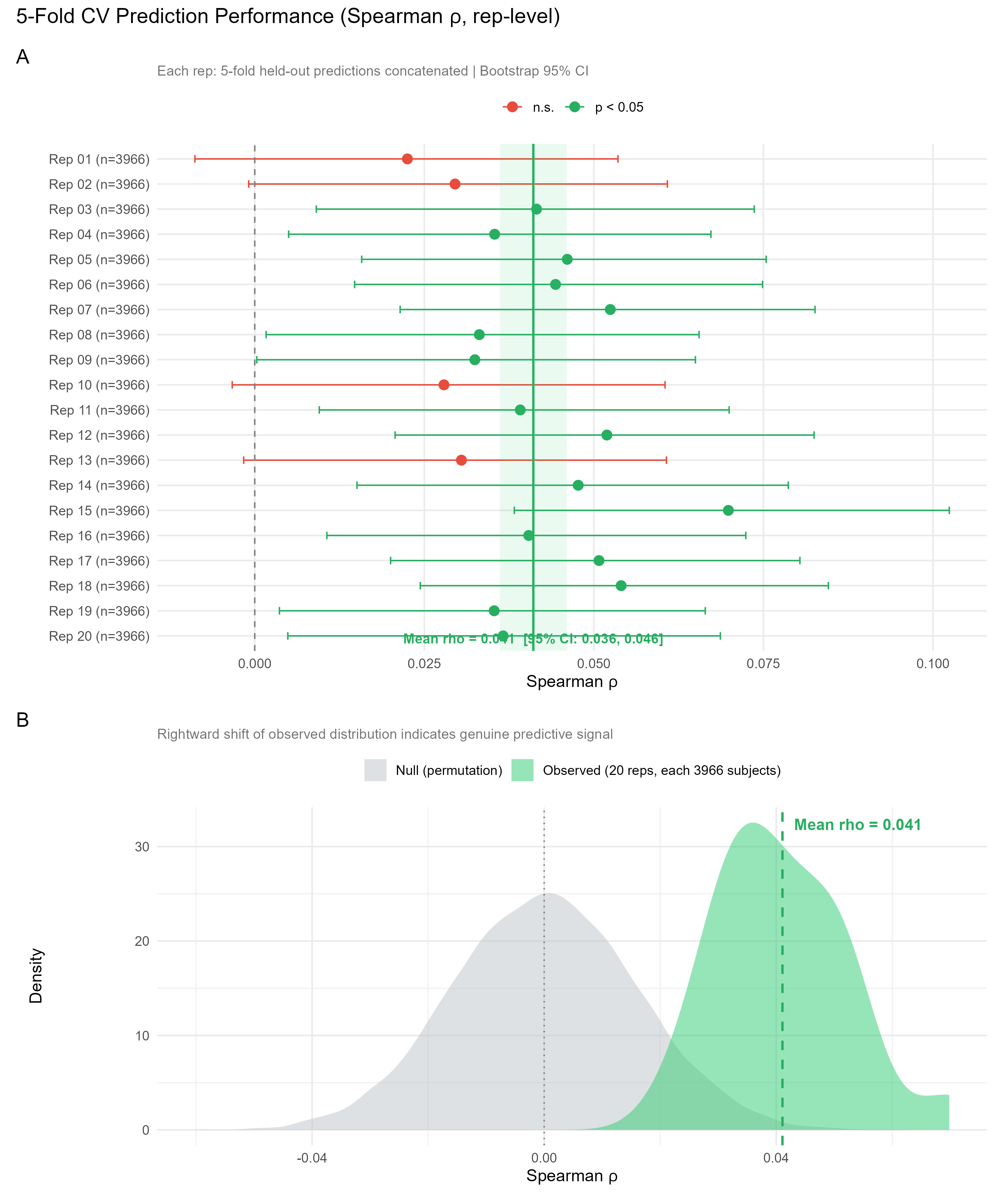


eFigure 7: Out-of-sample RDS-4 association strength across 20 repetitions of 5-fold stratified cross-validation. We evaluated out-of-sample association strength of RDS-4 depression symptom scores using components derived from the MCCAR+jICA decomposition. Within each repetition, held-out predictions from all 5 folds were concatenated (covering all 3,966 subjects exactly once), and a single correlation between predicted and actual RDS-4 scores was computed. (A) Forest plot showing the Spearman ρ and bootstrap 95% confidence interval (2,000 resamples) for each of the 20 repetitions. Green points indicate statistically significant predictions (p < 0.05); red points indicate non-significant results. The green vertical line and shaded band represent the overall mean ρ = 0.041 and its 95% CI [0.036, 0.046], which does not cross zero, confirming a consistent positive predictive signal. 16 of 20 repetitions (80%) reached statistical significance, far exceeding the 5% expected by chance. (B) Density plot comparing the distribution of 20 observed repetition-level ρ values (green) against a permutation-based null distribution (grey). The null was constructed by randomly shuffling the actual RDS-4 scores within each repetition 1,000 times (preserving the predicted values), yielding 20 x 1,000 = 20,000 null ρ values. This permutation procedure breaks the true association between predicted and actual scores while preserving the marginal distributions of both variables. The clear rightward separation of the observed distribution from the permutation null confirms that the predictive signal is genuine and not attributable to chance.

To ensure that the identified multimodal neuroimaging-plasma protein covariation component of depression (NeuroPro-Dep) reflects a stable population-level biological signature rather than an artifact of specific subject compositions or over-fitting, we conducted two levels of validation analysis. Specifically, we implemented a repeated 5-fold stratified cross-validation (CV) framework with 20 repetitions (100 total iterations), where the entire pipeline---including the MCCAR+jICA multimodal fusion and downstream linear regression modeling---was independently re-executed within each randomized 80% training fold and evaluated on a held-out 20% test set. Within each repetition, every subject appeared in the test set exactly once across the five folds, thereby covering all 3,966 subjects per repetition. Results were aggregated at the repetition level: stability correlations were averaged across the 5 folds within each repetition to yield 20 repetition-level estimates, and held-out predictions were concatenated across the 5 folds (covering all subjects exactly once) to compute a single correlation per repetition.

The first analysis focused on the spatial stability of multimodal features to examine the consistency of biological patterns across cross-validation iterations, with results detailed in eFigure 6. For each modality, we computed spatial correlations between the CV-derived and original NeuroPro-Dep spatial maps, considering both the full feature vectors and the core features (i.e., features with high absolute spatial |Z|-scores in the original NeuroPro-Dep component, as highlighted in Figure 2). The results indicate generally consistent spatial patterns across modalities. The Plasma Protein modality showed the highest stability, with a median spatial correlation of |r| > 0.92 for both full feature vectors and core signatures. Among neuroimaging modalities, core-feature (masked) correlations were higher than those of the full vectors: subcortical volume (median masked |r| = 0.94), functional connectivity (0.89), cortical thickness (0.86), structural connectivity (0.78), and cortical area (0.73) all exceeded the |r| = 0.7 benchmark, suggesting that the identified key brain alterations are broadly reproducible across independent sub-samples. Full-vector correlations for neuroimaging modalities were comparatively lower (median |r| = 0.55–0.64) but remained substantial given the high dimensionality and complexity of neuroimaging data.

The second analysis was designed to assess whether the identified components showed stable predictive utility for RDS-4 across the hold-out test sets, as shown in eFigure 7. Using Spearman rank correlation to minimize sensitivity to outliers, the distribution of 20 repetition-level correlation coefficients showed a significant positive shift compared to a permutation-based null distribution. The null was constructed by randomly shuffling actual RDS-4 scores within each repetition 1,000 times, yielding 20,000 null values. The observed mean Spearman rho = 0.041 (95% CI: [0.036, 0.046]) was entirely separated from the null, and 16 of 20 repetitions (80%) reached individual statistical significance (p < 0.05)---far exceeding the 5% expected by chance. Although effect sizes are modest in magnitude, the consistency across repetitions, the clear separation from the permutation null suggest a genuine, reproducible association between NeuroPro-Dep-derived features and depression symptom severity.

eFigure 8: Within-case validation of NeuroPro-Dep predictive capacity for symptom severity. Scatter plots illustrate the association between predicted and actual Recent Depressive Symptoms (RDS-4) scores exclusively within the depressed subgroup (ICD-10 diagnosis positive) of the UK Biobank validation cohort. A linear regression model was fitted using each modality's NeuroPro-Dep-derived features (loading scores and spatial back-reconstruction values) as predictors, and the correlation between predicted and observed RDS-4 scores was evaluated. Analysis was conducted for six modalities: cortical area (n = 2,530), functional connectivity (FC, n = 2,530), structural connectivity (SC, n = 2,530), cortical thickness (n = 2,530), subcortical volume (n = 2,530), and plasma proteins (n = 2,782; separate proteomic cohort). Point color reflects the local relative density estimated by kernel density estimation. Each panel displays the correlation coefficient (r), R^2^, 95% confidence interval, and associated p-value. Statistical significance is denoted as: * p < 0.05, ** p < 0.01, *** p < 0.001.

To rigorously address concerns regarding "double dipping" or potential bias introduced by using ICD-10 diagnostic labels as a reference signal during the multimodal fusion stage, we performed an independent within-case validation. By restricting the analysis to individuals already diagnosed with depression (ICD-10 label = 1), we effectively eliminated diagnostic label variance from the model. In this setting, any remaining predictive power for symptom severity must derive from genuine biological variations related to the clinical spectrum of the disorder rather than the binary supervision signal.

The results confirm that the neuroimaging-derived features of **NeuroPro-Dep** capture a continuous pathological spectrum beyond a simple diagnostic mirror. Specifically, structural connectivity (FC) demonstrated the strongest association (r = 0.06, $p=1\times{10}^{-4}$), followed by structural connectivity and cortical thickness. While the plasma protein modality did not reach significance in this patient-only subset (p = 0.3), its robust performance in the full validation cohort suggests it may serve primarily as a stable trait-marker for diagnosis. Overall, the persistent significance across all brain modalities within the depressed group provides strong evidence that the discovered multimodal patterns reflect objective biological substrates of depressive symptom severity.


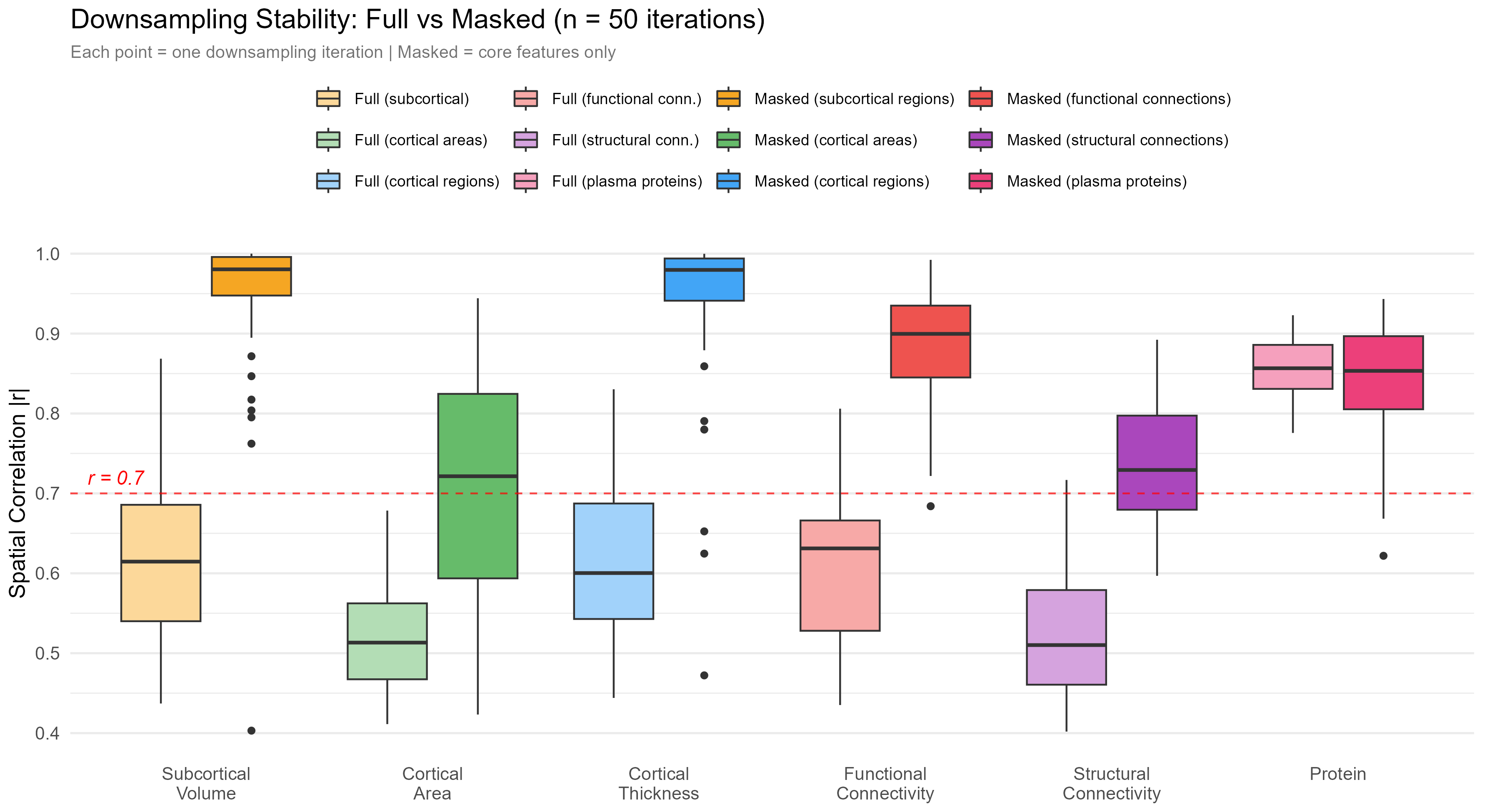


eFigure 9: Evaluation of component stability against case-control imbalance. To address concerns regarding the initial sample imbalance (360 depression cases vs. 3,606 healthy controls), we performed 50 independent iterations of 1:1 case-control down sampling. In each iteration, 360 controls were randomly selected to match the number of cases, and the entire MCCAR+jICA fusion pipeline was re-executed. The boxplots illustrate the absolute spatial correlation (|r|) between these down sampled components and the original discovery component (NeuroPro-Dep). Grey bars: Spatial correlation calculated using the full feature vectors of each modality. Colored bars: Spatial correlation restricted to masked core features (regions or proteins with high absolute loading Z-scores in the original component). The red dashed line at |r| = 0.7 serves as a benchmark for high spatial consistency. Modalities include Subcortical Volume, Cortical Area, Cortical Thickness, Functional Connectivity (FC), Structural Connectivity (SC), and Plasma Protein.

To ensure that the identified NeuroPro-Dep component is a robust biological signature and not an artifact of the initial case-control imbalance in our discovery cohort (360 cases vs. 3,606 controls), we conducted a rigorous down sampling sensitivity analysis. We repeatedly re-ran the complete multimodal fusion protocol 50 times, each time utilizing a randomly selected subset of 360 healthy controls to achieve a balanced 1:1 ratio with the patient group. The results demonstrate stability across all modalities, particularly within the core biological features. Specifically, the plasma protein modality maintained a median spatial correlation of $\left| r \right|>0.8$ with the discovery pattern, confirming the robustness of the proteomic signature regardless of control sample size. Within the neuroimaging modalities, the "masked" core features for subcortical volume and cortical thickness exhibited median correlations near $1.0$, indicating that the key anatomical alterations captured by NeuroPro-Dep are highly stable. While the correlations for the full feature vectors were lower—a predictable result given the background noise inherent in high-dimensional whole-brain data—they consistently remained robustly associated with the original discovery maps. Collectively, this analysis proves that the 360 depression cases provided sufficient statistical power to capture a reliable and stable multimodal pattern, and that the identified component is not purely driven by the disproportionately large control group.

**eMethods**

**Parameter Selection and Optimization for MCCAR+jICA**

We performed multimodal fusion using Multi-set Canonical Correlation Analysis with Reference plus Joint Independent Component Analysis (MCCAR+jICA), implemented in the Fusion ICA Toolbox (FIT, v2.0e) (Calhoun & Adali, 2009) under MATLAB R2024b. All parameters were specified in a batch configuration script and executed via ica_fuse_batch_file(). Below we describe the parameter selection process and justification for each key decision.

Dimensionality reduction (MCCAR stage). MCCAR was applied as the PCA variant (type_pca = 'mccar') to incorporate the reference signal into the dimensionality reduction. The number of principal components retained per modality group was set to [50, 50, 50, 50, 200] (cca_opts.numPC). For lower-dimensional modalities (subcortical volume, cortical area, cortical thickness, and functional connectivity), 50 PCs were retained, capturing over 95% of cumulative variance; for subcortical volume (20 features), this effectively preserves all variance. For higher-dimensional modalities (structural connectivity and plasma proteomics), 200 PCs were retained to adequately represent the richer variance structure prior to CCA. This configuration balances variance preservation against computational tractability in the subsequent CCA step. All remaining hyperparameters (e.g., regularization coefficients) were left at the FIT toolbox defaults, consistent with prior applications of this framework(Calhoun & Adali, 2009; Qi et al., 2018).

Independent component analysis (jICA stage). Ten independent components were extracted (numComp = 10) using the Infomax algorithm (Bell & Sejnowski, 1995), the default and most widely adopted ICA algorithm in the FIT framework for multimodal fusion (Calhoun & Adali, 2009; Sui et al., 2013). The choice of 10 components follows conventions established in prior multimodal fusion studies (Sui, Adali, Yu, Chen, & Calhoun, 2012; Sui et al., 2013) and balances sufficient granularity to capture distinct biological sources against the risk of over-decomposition. Higher model orders (20-30) were explored during pilot analyses but yielded additional components that either failed significance testing or showed poor stability.

To address the inherent stochasticity of ICA, we employed ICASSO (Himberg, Hyvärinen, & Esposito, 2004) with 10 repeated runs (num_ica_runs = 10). ICASSO clusters the resulting components across runs and selects centrotypes (the most representative component per cluster), providing a stability index (Iq) for each component. The reported NeuroPro-Dep component (IC #7) demonstrated high ICASSO stability.

Post-hoc significance testing. Component significance was assessed through a two-stage procedure. First, for each independent component, the mixing coefficients were compared between healthy and depression groups via two-sample t-tests across all six modalities; a component was considered significant only if all modalities reached p < 0.05. Second, a permutation test (10,000 iterations) was performed by correlating loadings with shuffled reference labels to estimate empirical p-values, guarding against chance findings.

**Modalities for Multimodal Fusion**

The data used for multimodal fusion were obtained from the UK Biobank and included several neuroimaging features, plasma proteins, and ICD-10 depression diagnosis labels. Subcortical volume data were derived from Category 190 using the ASEG template. Cortical surface area data were obtained from Category 197, field IDs 27334–27624, using the Destrieux template, while cortical thickness data were extracted from Category 197, field IDs 27408–27698, also based on the Destrieux template. Resting-state functional connectivity data were derived from field IDs 31015 and 31019, where cortical time series (field ID 31015, DK template) and subcortical time series (field ID 31019, Tian Subcortex S1 template) were concatenated to compute functional connectivity matrices using Pearson correlations. Structural connectivity data were obtained from field ID 31021, where the connectivity strength was quantified using the Fractional Anisotropy (FA) metric based on the aparc+Tian subcortex S1 template. Depression diagnosis labels were based on ICD-10 codes (F32 and F33), derived from field IDs 130894 and 130897. Plasma protein data were obtained from Category 1839.

The plasma protein data were sourced from the UK Biobank Pharma Proteomics Project consortium. Blood samples were collected in 9 mL EDTA vacutainers and processed into 850 μL aliquots containing EDTA plasma, buffy coat, and red blood cells. Plasma samples were stored at −80 °C before being transported on dry ice to Olink Analysis Service in Sweden. From April 2021 to January 2022, a proximity extension assay combined with Next-Generation Sequencing was employed to quantify 2,920 distinct proteins in parallel(Elliott, Peakman, & UK Biobank, 2008). After rigorous quality control procedures (details available at biobank.ndph.ox.ac.uk/ukb/ukb/docs/PPP_Phase_1_QC_dataset_companion_doc.pdf), proteins were assessed across four panels encompassing cardiometabolic, inflammatory, neurological, and oncological proteins. Additional details regarding sample selection, as well as Olink assay processing and quality control, have been documented in previous studies(Sun et al., 2023; Wik et al., 2021). Ultimately, 2,920 unique proteins were included in this study.

**Covariates**

During the data fusion stage, basic covariates, including sex, age, and head motion, were included in the model. For the plasma protein modality, additional covariates included sampling batch, fasting time, blood pressure, and the time difference between plasma protein sampling and brain imaging data collection, ensuring that variations in the interval between the two sampling sessions did not confound the relationship between proteins and other modalities in the fusion component.

In subsequent analyses—such as prediction, phenotype-wide association, polygenic risk score association, and future physical disease risk prediction—nuisance regression was applied to the features of each modality to mitigate the influence of covariates. For the brain imaging validation cohort, covariates included sex, age, the interaction between sex and age, the quadratic term of age, ethnicity, site, and body fat percentage. Additionally, head motion was regressed out for structural and functional connectivity, and total brain volume was regressed out for cortical surface area and thickness. For the plasma protein validation cohort, the same basic covariates were used, with the addition of blood pressure, fasting time, and sampling batch. In external dataset validation analyses, nuisance regression was similarly applied, using gender, age, the interaction between gender and age, the quadratic term of age, and race. For the SWU Depression cohort, race was not included as all participants were Han Chinese.

**Multimodal Data Preprocessing**

Before multimodal fusion, the plasma protein data underwent median imputation to maximize the use of rare protein data. For neuroimaging modalities, participants with excessive head motion were excluded from the functional connectivity and structural connectivity datasets. Subsequently, nuisance regression was applied to all modalities to account for basic covariates, including sex (field ID 31), age (field ID 21003; instance 0 for plasma protein modality, instance 2 for other neuroimaging modalities), ethnicity (field ID 21000), and site (field ID 54).

For the plasma protein modality, additional covariates were regressed out, including sampling batch (Category 1839, resource ID 1016), fasting time (field ID 74), blood pressure (field ID 4079), and the time difference between plasma protein sampling and brain imaging data collection (field ID 21003, instance 2 - instance 0). This adjustment ensured that variability in the time interval between the two sampling sessions did not confound the relationship between plasma proteins and other modalities in the fusion components.

**Thresholding of Spatial Maps Across NeuroPro-Dep Modalities**

After multimodal fusion identified NeuroPro-Dep, feature dimensions (S) for each modality were standardized, and specific thresholds (Z) were applied to derive spatial maps for each modality. As illustrated in Figure 2 of the main text, thresholds of |Z| > 2 were used for cortical surface area and thickness dimensions. For subcortical volume dimensions, the three regions with the highest |Z| values were selected, including the left and right hippocampus and the right thalamus. For structural and functional connectivity, thresholds of |Z| > 2 were applied to each edge, and the number of significant edges per brain region was calculated to generate ROI-wise spatial maps.

A similar method was applied to the plasma protein modality, as shown in Figure 5 of the main text, where a threshold of |Z| > 3 was used to identify a set of prominent plasma proteins.

**Linear back reconstruction**

Using a linear reverse reconstruction approach, the loadings of NeuroPro-Dep for each modality could be reconstructed in the UKB validation cohorts and external validation datasets, providing a third type of feature, as described in Equation 1(Qi et al., 2021).

$$A_{k}^{\text{ukbvalid}\text{/}\text{outervalid}}=X_{k}^{\text{ukbvalid}\text{/}\text{outervalid}}\cdot\left( S_{k}^{\text{explore}} \right)^{-1}$$

Here, $A_{k}^{\text{ukbvalid}\text{/}\text{outervalid}}$ represents the reconstructed loadings for a specific modality k in either the UKB validation cohort or external validation datasets.$X_{k}^{\text{ukbvalid}\text{/}\text{outervalid}}$ denotes the data for modality k in the UKB validation or external validation datasets, and $S_{k}^{\text{explore}}$ is the spatial map of NeuroPro-Dep for each modality derived from the discovery dataset.

**Association Analysis Using RDS-4 Scale**

The predictive model was tested separately for each modality. For each modality, a linear model was constructed using the three features mapped from the discovery cohort’s NeuroPro-Dep, as described in Equation 2.

$$RDS-4=\beta_{0}+{positive\_modal\_feature_{a}verage}_{i}\times\beta_{1}+{negative\_modal\_feature_{a}verage}_{i}\times\beta_{2}+A_{k}^{valid}\times\beta_{3}$$

Here, the positive and negative modality features were obtained by applying a predefined |Z|-threshold to the modality-specific feature-importance map ($S_{k}$) derived from NeuroPro-Dep. The ROIs (regions of interest) with values above or below the threshold were averaged to obtain the positive and negative features, respectively. Furthermore, using a linear reverse reconstruction approach, the loadings of NeuroPro-Dep for each modality could be reconstructed in the UKB validation cohorts and external datasets, providing a third type of feature.

**NeuroPro-Dep and Neurotransmitter Map Association Analysis**

To investigate the association between NeuroPro-Dep and neurotransmitter maps, we focused on the SC and FC modalities. For each modality, significant positive and negative edge maps were generated using the thresholds described earlier (|Z| > threshold). The cumulative number of positive and negative edges within each brain region (ROI) was then calculated.

These cumulative patterns of positive and negative changes for each brain region were analyzed using the JuSpace(Dukart et al., 2021) toolbox to assess their associations with 12 neurotransmitter PET maps.

**Validation of NeuroPro-Dep in External Datasets**

To evaluate the performance of NeuroPro-Dep in external datasets, we analyzed the association between the brain imaging modality and depressive symptoms using the HCP dataset and the SWU depression dataset. In the HCP dataset, the number depressive symptoms scale was used as the depression measure. Here, we selected four modalities available—functional connectivity (FC), cortical thickness (CT), cortical surface area (CSA), and subcortical volume (SV)—to jointly participate in the prediction. Using the same feature extraction method as applied to the UKB validation dataset, three NeuroPro-Dep-mapped features could be obtained for each modality. A total of 1,089 participants with complete data for all modalities and the depression scale were included. The validation models for the HCP and SWU Depression datasets are described in Equation 3.

$$Num depressive symptoms/HAMD=\beta_{0}+\beta_{1}\text{positive}\text{\_}\text{SV}\text{\_}\text{average}+\beta_{2}\text{negative}\text{\_}\text{SV}\text{\_}\text{average}+\beta_{3}A_{\text{SV}}+\beta_{4}\text{positive}\text{\_}\text{CSA}\text{\_}\text{average}+\beta_{5}\text{negative}\text{\_}\text{CSA}\text{\_}\text{average}+\beta_{6}A_{\text{CSA}}+\beta_{7}\text{positive}\text{\_}\text{CT}\text{\_}\text{average}+\beta_{8}\text{negative}\text{\_}\text{CT}\text{\_}\text{average}+\beta_{9}A_{\text{CT}}+\beta_{10}\text{positive}\text{\_}\text{FC}\text{\_}\text{average}+\beta_{11}\text{negative}\text{\_}\text{FC}\text{\_}\text{average}+\beta_{12}A_{\text{FC}}+\beta_{13}$$

For the SWU Depression dataset, the HAMD was employed to assess depression severity. The same modalities as those described for the HCP dataset were used, with the analysis restricted to individuals diagnosed with depression, resulting in a total of 242 participants.

**Identification of Directly Associated Proteins**

To analyze whether these significant proteins differ in biological pathways from proteins directly associated with depression diagnoses, we referred to methods from previous literature(Kang et al., 2025). Specifically, we predicted participants’ future depression risk using 2,920 plasma proteins. The prediction model employed a Cox proportional hazards regression framework implemented through the survival package in R (Therneau, until 2009), Elizabeth, & Cynthia, 2024). Depression diagnosis information was based on ICD-10 codes (F32, F33), with the onset date constrained to occur after plasma protein sampling. Participants were considered at risk for developing depression until they were diagnosed with a specific condition, lost to follow-up, deceased, or reached the last date of available data. Proteins with significant predictive capability in this model were categorized as proteins directly associated with depression diagnosis.

**Phenotype-Wide Association Analysis of NeuroPro-Dep**

We used the same linear model as employed for predicting RDS-4 scale to evaluate the associations between the plasma protein modality of NeuroPro-Dep and specific phenotypes, using the same plasma protein validation cohort. Specifically, the three features of the plasma protein modality were input into the linear model to generate predicted values for each phenotype. These predicted values were then input into phesant, a tool designed for phenotype-wide association analysis in the UKB dataset(Millard, Davies, Gaunt, Davey Smith, & Tilling, 2018). Phesant automatically selects the appropriate evaluation model based on the type of phenotype and ultimately returns the p-value for the association between the predicted and actual values of the phenotype. Given that complex phenotypes are significantly influenced by fundamental demographic characteristics, we additionally regressed out socioeconomic status and education level during the analysis to ensure the robustness of the identified associations. (Shen et al., 2025)

For phenotype selection, we utilized a wide range of UKB phenotype data, including variables related to social background, lifestyle, family history, cognitive function, mental health, physical measurements, medical conditions, and biomarkers. The selection criteria for phenotypes were as follows: first, all variables were sourced from the comprehensive set of UKB phenotype definitions provided by the phesant package; second, phenotypes were grouped into broad categories relevant to the research objectives, such as the aforementioned domains; finally, phenotypes were intersected with the plasma protein validation cohorts, with categories containing insufficient sample sizes excluded. These filtering steps were automated by the phesant package.

**Polygenic Risk Score Association Analysis of NeuroPro-Dep**

Polygenic risk score (PRS) data were obtained directly from the UK Biobank (Category 300) and included scores for various physical measurements and diseases. The association analysis with PRS was performed using the plasma protein validation cohort of NeuroPro-Dep. The method for establishing associations with the plasma protein modality of NeuroPro-Dep was consistent with the approach described in the phenotype analysis section.

**Association with Future Physical Disease Risk**

In this analysis, we used the Cox proportional hazards regression model to evaluate the association between the plasma protein modality loadings $A_{pro}^{ukbvalid}$ and the onset times of various physical diseases in the plasma protein validation cohort. For predicting the onset risk of various physical diseases, participants with disease onset dates prior to any modality data sampling were excluded to ensure the assessment focused on future disease risks. Participants were considered at risk for developing a particular disease until they were diagnosed with that disease, lost to follow-up, deceased, or reached the final date of available information. The survival analysis was performed using the survival package in R(Therneau et al., 2024).

**Detailed Statistical information**

The following provides a detailed report of the statistical results presented in the main text. In Figure 2, boxplots for each modality were analyzed using two-sample t-tests. For the cortical surface area modality, the results were t = 2, df = 3964, p = .04556, n = 3966. For the FC modality, t = 2.11, df = 3964, p = .03527, n = 3966. For the plasma protein modality, t = 2.77, df = 3964, p = .005553, n = 3966. For the SC modality, t = 2.34, df = 3964, p = .01953, n = 3966. For the cortical thickness modality, t = 2.62, df = 3964, p = .008789, n = 3966. For the subcortical volume modality, t = 3.14, df = 3964, p = .001719, n = 3966. In Figure 3, the prediction of RDS-4 scale was evaluated using the Pearson correlation between predicted and actual values, calculated with the cor.test() function in R. For the UKB brain imaging validation cohort, the subcortical volume yielded r = .02, df = 28643, p = 2.548e-05, n = 28645, the cortical surface area yielded r = .03, df = 28643, p = 1.954e-08, n = 28645, the cortical thickness yielded r = .02, df = 28643, p = .0004823, n = 28645, the FC modality yielded r = .03, df = 28643, p = 1.268e-08, n = 28645, and the SC modality yielded r = .02, df = 28643, p = .001101, n = 28645. For the UKB plasma protein validation cohort, the results were r = .02, df = 36807, p = .0001004, n = 36809. In Figure 4, external dataset validation was also performed using the cor.test() function. For the HCP dataset, the results were r = 0.1, df = 1051, p = .0007509, n = 1084, with 31 subjects excluded due to missing data. For the SWU depression dataset, the results were r = 0.24, df = 215, p = .0002936, n = 242, with 25 subjects excluded due to missing data. The detailed results of the polygenic risk score association analysis, the phenome-wide association study, and the prediction of future physical disease onset are provided in the attachment files.

**eResults**

**Association of plasma protein modality in NeuroPro-Dep with related factors**

To further explore the associations between NeuroPro-Dep and various phenotypic domains, such as disease history, environment, lifestyle, physical health, and cognitive function, we utilized the phesant package(Millard et al., 2018)—a tool for conducting comprehensive phenome-wide association analyses in UKB. Using the same linear model construction and participant selection approach as employed for predicting RDS-4 depression scores, we examined the relationships between the plasma protein modality of NeuroPro-Dep and phenotypes.

As shown in main text Figure 6(a), we generated a Manhattan plot illustrating the associations between the plasma protein modality and phenotypes. The results revealed significant associations with environmental factors (e.g., nitrogen dioxide pollution, p = 3.06 × 10⁻¹⁹***; PM10, p = 2.2 × 10⁻³³***), socioeconomic status (e.g., housing score, p = 1.02 × 10⁻¹¹***; income score, p = 2.3 × 10⁻⁹***), cognitive function (e.g., prospective memory, p = 2.1 × 10⁻⁵***), mental health (e.g., alcohol drinking frequency, p = 8 × 10⁻²⁸***; frequency of tiredness or lethargy, p = 8.03 × 10⁻⁵***), blood biomarkers (e.g., white blood cell count, p < 1 × 10⁻³⁰⁰***; triglycerides, p < 1 × 10⁻³⁰⁰***), body measurements (e.g., impedance of the left leg, p = 4.4 × 10⁻²⁶***; impedance of the right leg, p = 1.5 × 10⁻²⁸***), and, notably, physical diseases (e.g., diabetes diagnosed by a doctor, p = 2.4 × 10⁻²¹***; initiation of insulin treatment within one year of diabetes diagnosis, p = 3.5 × 10⁻¹³***; age at diabetes diagnosis, p = 1.7 × 10⁻¹⁰***).

Furthermore, we conducted association analyses between the plasma protein modality of NeuroPro-Dep and polygenic risk scores (PRS) for various physical measurements and diseases. As shown in main text Figure 6(b), significant associations were observed with polygenic risk scores for high-density lipoprotein cholesterol (HDL, p = 3.9 × 10⁻¹⁴***), BMI (p = 1.5 × 10⁻¹⁰***), type 2 diabetes (p = 1.37 × 10⁻⁹***), and multiple sclerosis within the neurological disease category (p = 5.7 × 10⁻⁴***).

These findings further underscore the close connections between the plasma protein modality of NeuroPro-Dep and physical diseases, lifestyle factors, and environmental influences, as demonstrated by the phenotypic and polygenic risk score analyses.

**Association Strength of the Plasma Protein Modality of NeuroPro-Dep for Future Physical Disease Onset**

Phenome-wide association analysis based on cross-sectional data confirmed the extensive associations between NeuroPro-Dep and physical diseases. To explore the potential impacts of depression-related protein alterations on the future onset of various physical diseases, we analyzed longitudinal disease follow-up data. Using Cox proportional hazards regression models with the loadings of the plasma protein modality of NeuroPro-Dep as predictors, we attempted to forecast future diagnoses of physical diseases after the time of plasma protein sampling. Disease outcomes were defined based on all ICD-10 physical disease diagnoses available in the UKB dataset.

As shown in main text Figure 6(c), we identified both protective and risk-related effects of the plasma protein modality of NeuroPro-Dep on various physical diseases. Protective effects were observed for conditions such as asthma (J45, HR = .013, p = 1.57 × 10⁻⁶***), lipoprotein metabolism and dyslipidemia (E78, HR = 0.10, p = .001***), and kidney stones (N20, HR = 9.2 × 10⁻⁴, p = .002**). However, the plasma protein modality of NeuroPro-Dep more commonly acted as a risk factor for physical diseases, including cardiovascular diseases such as hypertensive nephropathy (I12, HR = 2.3 × 10¹¹, p = 4.5 × 10⁻⁹***), atrial fibrillation (I48, HR = 1 × 10³, p = 1.3 × 10⁻⁷***), heart failure, myocardial infarction, deep vein thrombosis, and varicose veins. Significant associations were also observed for kidney diseases, including chronic kidney disease (N18, HR = 3.3 × 10³, p = 6.79 × 10⁻⁹***) and renal insufficiency (N19, HR = 3.1 × 10⁸, p = 2.77 × 10⁻⁷***). Metabolic diseases such as pancreatic endocrine disorders (E16, HR = 1.7 × 10⁶, p = .00026***) and other disorders involving fluid and electrolyte balance (E87, HR = 625, p = 8.37 × 10⁻⁵***) were also significantly associated. Additionally, the modality was linked to other chronic diseases, including fundus disorders potentially related to diabetes or hypertension (H36, HR = 1.2 × 10⁶, p = 7.77 × 10⁻⁵***).

**Reference:**

Bell, A. J., & Sejnowski, T. J. (1995). An Information-Maximization Approach to Blind Separation and Blind Deconvolution. *Neural Computation*, *7*(6), 1129–1159. https://doi.org/10.1162/neco.1995.7.6.1129

Calhoun, V. D., & Adali, T. (2009). Feature-Based Fusion of Medical Imaging Data. *IEEE Transactions on Information Technology in Biomedicine*, *13*(5), 711–720. https://doi.org/10.1109/TITB.2008.923773

Dukart, J., Holiga, S., Rullmann, M., Lanzenberger, R., Hawkins, P. C. T., Mehta, M. A., … Eickhoff, S. B. (2021). JuSpace: A tool for spatial correlation analyses of magnetic resonance imaging data with nuclear imaging derived neurotransmitter maps. *Human Brain Mapping*, *42*(3), 555–566. https://doi.org/10.1002/hbm.25244

Elliott, P., Peakman, T. C., & UK Biobank. (2008). The UK Biobank sample handling and storage protocol for the collection, processing and archiving of human blood and urine. *International Journal of Epidemiology*, *37*(2), 234–244. https://doi.org/10.1093/ije/dym276

He, R., Zheng, R., Zheng, J., Li, M., Wang, T., Zhao, Z., … Ning, G. (2023). Causal Association Between Obesity, Circulating Glutamine Levels, and Depression: A Mendelian Randomization Study. *The Journal of Clinical Endocrinology and Metabolism*, *108*(6), 1432–1441. https://doi.org/10.1210/clinem/dgac707

Himberg, J., Hyvärinen, A., & Esposito, F. (2004). Validating the independent components of neuroimaging time series via clustering and visualization. *NeuroImage*, *22*(3), 1214–1222. https://doi.org/10.1016/j.neuroimage.2004.03.027

Kang, J., Yang, L., Jia, T., Zhang, W., Wang, L.-B., Zhao, Y.-J., … Cheng, W. (2025). Plasma proteomics identifies proteins and pathways associated with incident depression in 46,165 adults. *Science Bulletin*, *70*(4), 573–586. https://doi.org/10.1016/j.scib.2024.09.041

Millard, L. A. C., Davies, N. M., Gaunt, T. R., Davey Smith, G., & Tilling, K. (2018). Software Application Profile: PHESANT: a tool for performing automated phenome scans in UK Biobank. *International Journal of Epidemiology*, *47*(1), 29–35. https://doi.org/10.1093/ije/dyx204

Qi, S., Calhoun, V. D., van Erp, T. G. M., Bustillo, J., Damaraju, E., Turner, J. A., … Sui, J. (2018). Multimodal Fusion With Reference: Searching for Joint Neuromarkers of Working Memory Deficits in Schizophrenia. *IEEE Transactions on Medical Imaging*, *37*(1), 93–105. https://doi.org/10.1109/TMI.2017.2725306

Qi, S., Schumann, G., Bustillo, J., Turner, J. A., Jiang, R., Zhi, D., … Whelan, R. (2021). Reward Processing in Novelty Seekers: A Transdiagnostic Psychiatric Imaging Biomarker. *Biological Psychiatry*, *90*(8), 529–539. https://doi.org/10.1016/j.biopsych.2021.01.011

Shen, C., Zhang, R., Yu, J., Sahakian, B. J., Cheng, W., & Feng, J. (2025). Plasma proteomic signatures of social isolation and loneliness associated with morbidity and mortality. *Nature Human Behaviour*. https://doi.org/10.1038/s41562-024-02078-1

Sui, J., Adali, T., Yu, Q., Chen, J., & Calhoun, V. D. (2012). A review of multivariate methods for multimodal fusion of brain imaging data. *Journal of Neuroscience Methods*, *204*(1), 68–81. https://doi.org/10.1016/j.jneumeth.2011.10.031

Sui, J., He, H., Pearlson, G. D., Adali, T., Kiehl, K. A., Yu, Q., … Calhoun, V. D. (2013). Three-way (N-way) fusion of brain imaging data based on mCCA   +   jICA and its application to discriminating schizophrenia. *NeuroImage*, *66*, 119–132. https://doi.org/10.1016/j.neuroimage.2012.10.051

Sui, J., Qi, S., van Erp, T. G. M., Bustillo, J., Jiang, R., Lin, D., … Calhoun, V. D. (2018). Multimodal neuromarkers in schizophrenia via cognition-guided MRI fusion. *Nature Communications*, *9*(1), 3028. https://doi.org/10.1038/s41467-018-05432-w

Sun, B. B., Chiou, J., Traylor, M., Benner, C., Hsu, Y.-H., Richardson, T. G., … Whelan, C. D. (2023). Plasma proteomic associations with genetics and health in the UK Biobank. *Nature*, *622*(7982), 329–338. https://doi.org/10.1038/s41586-023-06592-6

Therneau, T. M., until 2009), T. L. (original S.->R port and R. maintainer, Elizabeth, A., & Cynthia, C. (2024). *survival: Survival Analysis*. Retrieved from https://cran.r-project.org/web/packages/survival/index.html

Wik, L., Nordberg, N., Broberg, J., Björkesten, J., Assarsson, E., Henriksson, S., … Lundberg, M. (2021). Proximity Extension Assay in Combination with Next-Generation Sequencing for High-throughput Proteome-wide Analysis. *Molecular & Cellular Proteomics: MCP*, *20*, 100168. https://doi.org/10.1016/j.mcpro.2021.100168
